# Evolution of behavioral flexibility and the forming and breaking of habits

**DOI:** 10.1093/evlett/qrag024

**Published:** 2026-06-19

**Authors:** Olof Leimar, Sasha R X Dall, Peter Hammerstein, Alasdair I Houston, Bram Kuijper, John M McNamara

**Affiliations:** Department of Zoology, Stockholm University, Stockholm, Sweden; Centre for Ecology and Conservation, University of Exeter, Penryn, United Kingdom; Institute for Theoretical Biology, Humboldt University Berlin, Berlin, Germany; School of Biological Sciences, University of Bristol, Bristol, United Kingdom; Centre for Ecology and Conservation, University of Exeter, Penryn, United Kingdom; School of Mathematics, University of Bristol, Bristol, United Kingdom

**Keywords:** exploration–exploitation trade-off, behavioral flexibility, multitasking, reinforcement learning, multi-armed bandits

## Abstract

The formation of habits, whereby learnt actions come to be performed automatically with repetition and practice, is a well-established focus for studies in psychology. This contrasts with evolutionarily motivated studies of learning, which typically view behavior as either learnt or fixed, to elucidate the ecological conditions where each predominates. Here, we envisage habit formation functioning to free up limited mental resources (e.g., attention), potentially improving an individual’s ability to multitask. As an ecologically relevant case, we investigate exploration–exploitation in foraging under predation risk. In our model, a forager does not know the quality of feeding options, but can learn from the rewards they give. When the environment occasionally changes, individuals can attend to exploration of feeding options in the new conditions. The options can then become habitually exploited, freeing attention for antipredatory vigilance. Via evolutionary simulations, we show that evolutionarily stable forming and breaking of foraging habits can substantially reduce mortality from predation, without drastically reducing foraging success, when environmental conditions remain stable enough between changes. We identify factors promoting habit formation, such as the repetition of actions that yield predictable rewards, and also discuss the role of habits more generally, including their relation to ideas about bounded rationality in theories of decision-making. In conclusion, we argue that exploration–exploitation strategies involving the forming and breaking of habits are a type of behavioral flexibility that is likely to be selected for under a range of ecological conditions.

## Teaser

The forming and breaking of habits has long been a focus of study in psychology, as well as appearing in many discussions about human behavior. Surprisingly, the topic has been virtually absent from evolutionary studies of learning in non-human animals, despite featuring prominently in Darwin’s discussion of the evolution of species-specific behaviors. Our aim is to change this, by theoretically investigating a possible evolutionary explanation for habits, namely that habits enhance an individual’s ability to multitask. We model the combination of foraging and antipredator vigilance, which is important for many animal species in their natural environments. We conclude that the forming and breaking of habits can promote behavioral flexibility, helping animals to achieve important tasks in different environments.

## Introduction

Habits, as studied in experimental psychology, are modes of behavior where individuals come to perform actions as more-or-less automatic responses, triggered by stimuli or circumstances they repeatedly encounter. The formation of habits is typically guided by goal-directed learning processes (Bouton, 2021, 2024; Buabang et al., 2025; Dolan & Dayan, 2013; Miller et al., 2019), making it a common feature of human task learning. Indeed, habit learning mechanisms seem to be conserved across the mammalian species that have been subject to laboratory reinforcement learning research (Wood & Rünger, 2016) and are suggested to be shared by all vertebrates (Grillner, 2025; Grillner & Robertson, 2016). Habits also figured prominently in Darwin’s thinking about the possible origin of species-specific behaviors (Gordillo-García, 2016; Portera & Mandrioli, 2021; see Appendix A). Despite these early ideas, behavioral and evolutionary ecologists typically consider the evolutionary stability of learning strategies compared to innate, fixed behavioral responses (Dridi & Lehmann, 2016; Kozielska & Weissing, 2024). The continuum between learnt, habitual, and fixed behavior instead argues for a more nuanced view of learnt behavior. Here, we suggest that the forming and breaking of habits represent important types of behavioral flexibility and deserve to be taken into account in evolutionary investigations of animal behavior.

An important property of habits is that, in terms of mental resources, they are efficient ways of performing tasks, by letting actions be guided reflexively by stimuli and contexts (Buabang et al., 2025; see Appendix B for explanations of terminology). In contrast to non-habitual behavior, which involves continuous learning and updating, habitual task performance enables individuals to free cognitive resources like attention to devote to other concerns (Bouton, 2021; Buabang et al., 2025). It is generally recognized that limited attention can constrain multitasking (Dukas, 2002, 2004). Here, we use evolutionary simulations to show that habit-enhanced multitasking can be selectively favored, by letting animals efficiently combine foraging and vigilance against predators. Our work is a further development of the study of foraging–vigilance trade-offs, which has long been a central topic in behavioral ecology (Beauchamp, 2015; Houston et al., 1993; Milinski & Heller, 1978).

To analyze the forming and breaking of habits, we use the idea of a trade-off between exploration and exploitation. This trade-off figures prominently in the fields of psychology, behavioral ecology, and machine learning (Ajuwon et al., 2025; Biswas et al., 2023; Cohen et al., 2007; Daw et al., 2006; Mehlhorn et al., 2015; Sutton & Barto, 2018), but there is no consensus about the cognitive mechanisms that control such behavior. Our approach is to assume that transitions between exploration and exploitation correspond to the forming of new habits and the breaking of old habits (Buabang et al., 2025; Dolan & Dayan, 2013; Miller et al., 2019; Wood & Neal, 2007; Wood & Rünger, 2016).

We examine situations where the reward distributions of different feeding options are unknown but remain constant over a period of time, after which they can change. In nature, a change could for instance be due to a new season or moving to a new location, and there could be several such environmental changes over an individual’s lifetime. If each successive environment is constant for long enough, it would be advantageous to first learn about the feeding options and then to become habitual in foraging, thus balancing the attentional demands of foraging and vigilance. Such situations could favor the evolution of psychological mechanisms for the forming and breaking of habits. It is our aim to establish the conditions under which these mechanisms are advantageous, by examining cases where exploration and learning influence predation risk, as well as cases with little or no influence. We also investigate factors influencing the speed of habit formation and illustrate situations where habits should not form.

In addition to changes in rewards, individuals can perceive other cues indicating new environments. These cues could be signs of seasonal change, or that an individual has reached a new location. There are psychological studies investigating the effects of cues of changes of context (Bouton, 2021, 2024; Buabang et al., 2025; Racey et al., 2011; Wood et al., 2005). In our modeling, we assume that such additional cues of change are available.

The elements of our models include psychological mechanisms of exploration and learning, as well as rules for the forming and breaking of habits. For exploration, we use traditional action-value learning, but modified such that the initial estimated values of options can be high (Sutton & Barto, 2018). We refer to the approach as optimistic exploration (OE). This approach is well established in theoretical work on reinforcement learning (Berger-Tal & Avgar, 2012; Brafman & Tennenholtz, 2002; Dey et al., 2024; Lobel et al., 2022; Steyvers et al., 2009; Strehl et al., 2006; Sutton & Barto, 2018) and has the advantage of a close correspondence to traditional assumptions about learning in experimental psychology. For comparison, in the supplements we also show results with the so-called Thompson sampling (TS) (Russo et al., 2018; Thompson, 1933) as the exploration strategy. TS is a Bayesian statistical approach that is argued to show good performance in some situations (Russo et al., 2018), but it is not known if it is used by real animals. Both OE and TS originated in the study of multi-armed bandits (Russo et al., 2018; Sutton & Barto, 2018).

To implement habit formation, we use the well-established idea in cognitive modeling (Miller et al., 2019; Wilson & Collins, 2019) that habits are characterized by consistency of choice, i.e. the tendency to choose a given action in a given situation. In our simulations, we express the consistency of choice as a running estimate of the recent frequency of the currently used action. We assume that as an individual’s consistency of choice passes a threshold, the individual switches to habitual foraging, at the same time increasing its vigilance against predators. For the reverse transition from habitual foraging to exploration, we assume that individuals can evolve to respond to a cue of a new environment, by resetting the attention to learning about foraging options, including restarting OE. We also study an alternative mechanism for detecting a change of environment, for which individuals break a habit after experiencing a sequence of low rewards from their habitual choices.

In the following, we illustrate the kinds of environmental volatility we investigate and show results from individual-based evolutionary simulations of the performance of exploration–exploitation strategies. We find that the forming and breaking of foraging habits has the potential to significantly reduce mortality from predation, without drastically reducing foraging success. We therefore argue that there is likely to be substantial natural selection for this form of behavioral flexibility.

## Methods

Here, we give an overview of the models we analyze, including the type of environments we study and the strategies individuals use in these environments. The strategies have parameters, and some of these parameters are assumed to be genetically determined traits. The evolution of these traits is studied in individual-based simulations, where the reproductive success is taken to be proportional to the total amount of reward an individual accumulates over its lifetime, up to death by predation or, otherwise, over the total number of trials assumed for the model. See Supplementary Tables S1 and S2 for equilibrium trait values and Supplementary Table S3 for notation.

### Distribution of rewards from feeding options

The mean values $\bar{R}_{k}$ of options vary between environments (see Figure 1A for an example). Each mean value $\bar{R}_{k}$ is independently drawn from a log-normal distribution (ensuring positive values) with a log-scale mean $\rho _0$ and log-scale SD $\sigma _0$. In the examples in the main text $\rho _0 = 0$ and $\sigma _0 = 1$. As is illustrated in Figure 1B, there is also random variation in the rewards from a particular feeding option. We assume that this variation is log normally distributed. Thus, for option *k*,


(1) \begin{eqnarray*}
R_{k}(t) = \exp \bigl ( \rho _{k} + z(t) \bigr ) \mathrm{,}
\end{eqnarray*}

where the *z(t)* are independent and normally distributed with mean zero and standard deviation $\sigma _R$ and with $\rho _{k}$ such that $\exp (\rho _{k} + \sigma _R^2 / 2) = \bar{R}_{k}$, where $\bar{R}_{k}$ is the mean reward from the option. This gives $\rho _{k} = \log (\bar{R}_{k}) - \sigma _R^2 / 2$. As illustrated in Figure 1B, we use $\sigma _R = 0.25$ in some of our examples. We sometimes use the notation *R* to mean $R_{k}(t)$ for the chosen option *k* in trial *t*.

### Predation risk and attention dynamics

In principle, individuals could pay attention to and learn about certain stimulus dimensions or features while ignoring others, but as a simplification we consider all learning about feeding options as regulated in an on/off fashion. Attention is indicated by a 0/1 variable *a*, with *a(t) = 1* in trial *t* if the individual is exploring and learning in that trial (using either OE or TS). The rate of predation in trial *t*, as a function of attention, is then


(2) \begin{eqnarray*}
\mu _\mathrm{pred}(t) = \mu _0 + \mu _1 a(t) \mathrm{.}
\end{eqnarray*}

For our analyses in the main text we have used $\mu _0 = 0.00016$ and $\mu _1 = 0.00032$, except for cases of no influence of attention on predation risk, where we use $\mu _1 = 0.0$. Over a total of 1,000 trials, having these values of $\mu _0$ and $\mu _1$ gives survival probabilities of 0.62 with full attention to exploration and 0.85 for pure habitual foraging. This means that habit formation allows a forager to decrease predation mortality by at most around 60% of its value for full attention to exploration.

For deciding when to turn off attention and become habitual, we assume that while learning an individual keeps track of its consistency of choice $h_k$ for each feeding option, as follows:


(3) \begin{eqnarray*}
h_{k}(t + 1) = h_{k}(t) + \alpha _h \bigl ( 1 - h_{k}(t) \bigr )
\end{eqnarray*}

for the chosen option *k* in trial *t* and


(4) \begin{eqnarray*}
h_{l}(t + 1) = h_{l}(t) + \alpha _h \bigl ( 0 - h_{l}(t) \bigr )
\end{eqnarray*}

for each non-chosen option *l ≠ k*. The $h_k$ can be thought of as measures of “habit strength,” satisfying $0 \le h_k < 1$, and $\alpha _h$ is a habit strength update rate. We sometimes write consistency of choice to mean the consistency $h_k$ of the chosen option on a given trial. This form of consistency of choice has been used previously (Miller et al., 2019) and has also been referred to as a “choice kernel” (Wilson & Collins, 2019). In our model, an individual sets *a(t)* to zero, thus turning off attention to exploration in trial *t*, if


(5) \begin{eqnarray*}
h_k(t) > \hat{h}
\end{eqnarray*}

for the chosen option *k*, in which case *k* becomes the habitual choice. The threshold $\hat{h}$ is a genetically determined trait and, in order to allow evolution toward no habit formation, we allow values $\hat{h} > 1$.

#### Cues of environmental change

We assume that, on the first trial of a potentially new environment, an individual observes an environmental cue, indicating whether the environment has changed. For most of our results, including those in the main text, we use reliable cues, i.e., cues such that when observing the cue, it is essentially certain that there is a new environment. In such a case, an individual then resets its state at the start of each new environment, by turning on attention and making a fresh start on learning about the feeding options. In the supplements, we give details of how we have implemented cues of change, and also give results for cues that are less reliable indicators of change.

Our assumption about cues of change is that they are very easy for a forager to detect, requiring almost no extra attention. This is likely to be the case for changes such as moving to a new place or a seasonal change, but it need not be the case for all conceivable cues of change. In the supplements we also investigate an alternative mechanism for detection of environmental change, in the form of an estimate of recent rewards going below a threshold.

### Optimistic exploration

We use action-value learning (Sutton & Barto, 2018) for the exploration part of the strategy, with the added element that the starting estimated values of the feeding options can be relatively high (“optimistic initial values”; Sutton & Barto, 2018). This is essentially a version of classical Rescorla–Wagner learning (Rescorla & Wagner, 1972), adapted to operant conditioning.

An individual has an estimate $w_{k}(t)$ of the reward value of choosing option *k* in trial *t*, and the starting value is assumed to be $w_{k}(0) = w_0$. Ideally, for OE, $w_0$ should be higher than any of the actual mean reward values of options in the current environment (cf. Figure 1C). If option *k* is chosen in trial *t*, the individual updates its estimate to $w_{k}(t + 1)$, where


(6) \begin{eqnarray*}
w_{k}(t + 1) = w_{k}(t) + \alpha _w \, \bigl ( R_{k}(t) - w_{k}(t) \bigr ) \mathrm{.}
\end{eqnarray*}

Here, $\alpha _w$ is the learning rate, for simplicity assumed to be constant over trials and the same for all options, and $R_{k}(t)$ is the reward perceived by the individual from interacting with a stimulus of type *k* at time *t*. The quantity


(7) \begin{eqnarray*}
\delta _{k}(t) = R_{k}(t) - w_{k}(t)
\end{eqnarray*}

is referred to as the prediction error and is the difference between the reward $R_{k}(t)$ currently experienced by the individual and its prior estimate $w_{k}(t)$ of the reward. The change in the estimated feature value $w_{k}$ in equation 6 is thus the learning rate $\alpha _w$ times the prediction error, and tends to move the estimate toward a “true value.”

#### Choice of options

For the choice of option we follow the standard approach by Sutton and Barto (2018) for action-value learning. In each trial of OE, an individual chooses the option with the highest estimated value $w_{k}$, except that in a proportion *ε* of cases the individual chooses a random option. This procedure is often referred to as an *ε*-greedy method of selecting actions (Sutton & Barto, 2018). The proportion *ε* introduces a certain amount of exploration (we have used *ε = 0.01*), but it should be noted that the possibility of initial optimistic estimated values is the most important feature of exploration in the model. If more than one option has the same maximal value, which will be the case at the start of OE, we assume that an individual simply selects the first of these.

#### Genetically determined traits

For evolutionary analysis, we assume that certain of the learning parameters are evolving traits. For OE, the initial value $w_0$ and the learning rate $\alpha _w$ are such traits. In addition to these, there are traits $\alpha _h$, $\hat{h}$ for the forming of habits, as described above. For OE, we implemented $\alpha _h$ as a product $\alpha _h = \alpha _0 \times \hat{h}$, with $0 \le \alpha _0 < 1$ giving how big a proportion the update rate is of the threshold $\hat{h}$. The reason for the implementation is that it reduces an otherwise rather strong positive correlation between $\alpha _h$ and $\hat{h}$ over evolutionary time. In the supplements, we also describe traits for detecting environmental change.

### Habitual foraging

For both OE and TS foragers, we assume that habitual foraging means that the last choice before the transition is repeated. The consistency of choice $h_{k}(t)$ stays constant during habitual foraging, and the attention variable *a(t)* in equation 2 is equal to zero, corresponding to increased vigilance.

### Order of events in a trial

In a trial, the following occurs: (*i*) check for environmental cue indicating habit breaking and resetting of exploration; (*ii*) risk of predation, following equation 2; (*iii*) choice of feeding option; (*iv*) reward; (*v*) if attention to exploration is on, update consistency of choice; (*vi*) for OE with attention on, perform learning updates; and (*vii*) if attention to exploration is on, check if it should be turned off.

### Evolutionary simulations

A simulated population has non-overlapping generations. For computational efficiency (multithreading) in evolutionary simulations, the population was split into groups of size 10, with 400 groups, making up a total of 4,000 individuals. Individuals are sexually reproducing, with one diploid locus for each trait, with additive alleles. For simplicity, we assume that individuals are hermaphrodites, mating is random across the entire population, and the different loci are unlinked. Also for simplicity, we assume that reproductive success accumulates proportional to collected rewards over all trials (so that an individual dying from predation still has reproductive success from its rewards collected until death; other assumptions about the life history, such as discrete reproductive seasons, are of course possible).

Evolutionarily stable trait values were obtained by first running simulations over several 100,000 generations, recording the mean trait values every 1,000 generations, until there were no systematic changes. Equilibria were then determined by running simulations for another 1,000,000 generations, computing mean trait values from values recorded every 1,000 generations. The data for the figures were then obtained by simulating a single generation with these mean trait values, randomly generating environments for each of 4,000 individuals, in order to achieve maximum sampling of environmental variation.

Simulations were implemented as C++ programs, with separate programs for OE and TS. The genetically determined traits are $w_0$, $\alpha _w$, $\alpha _h$, $\hat{h}$ for OE, and $\alpha _h$, $\hat{h}$ for TS (with additional habit-breaking traits described in the supplements). We implemented mutation as potentially occurring for each allele of a newly formed individual, with probability 0.002 and normally distributed mean-zero mutational increments. The standard deviation of the increments was adjusted to the range of variation of a given trait.

## Results

Figure 1 shows a situation that could promote the forming and breaking of habits. A forager is faced with a number of feeding options (we use eight feeding options). Environments are randomly generated, in the sense that the mean reward for each option is independently drawn from a log-normal distribution (Figure 1A). In each of a number of foraging trials, there is a choice of feeding option to use in that trial. Each feeding option delivers positive, log-normally distributed rewards, with a certain mean and (log-scale) random variation around this mean (Figure 1B). In our analyses we assess performance over two or more successive environments. Ideally, in such situations a forager should explore to find the highest yielding option in an environment. The intuition behind OE is sketched in Figure 1C. By starting with high initial estimates of reward rates, which then are reduced through the sampling of options, a forager selecting the option with currently highest estimated value is incentivized to sample all options and, in the ideal case, eventually to focus on the one with the highest expected reward. If a sufficiently thorough exploration can be achieved early in the period of an environment, and foraging then becomes habitual, the cost of increased predation risk from reduced vigilance during exploration and learning can be fairly small (Figure 1D).

**Figure 1.**
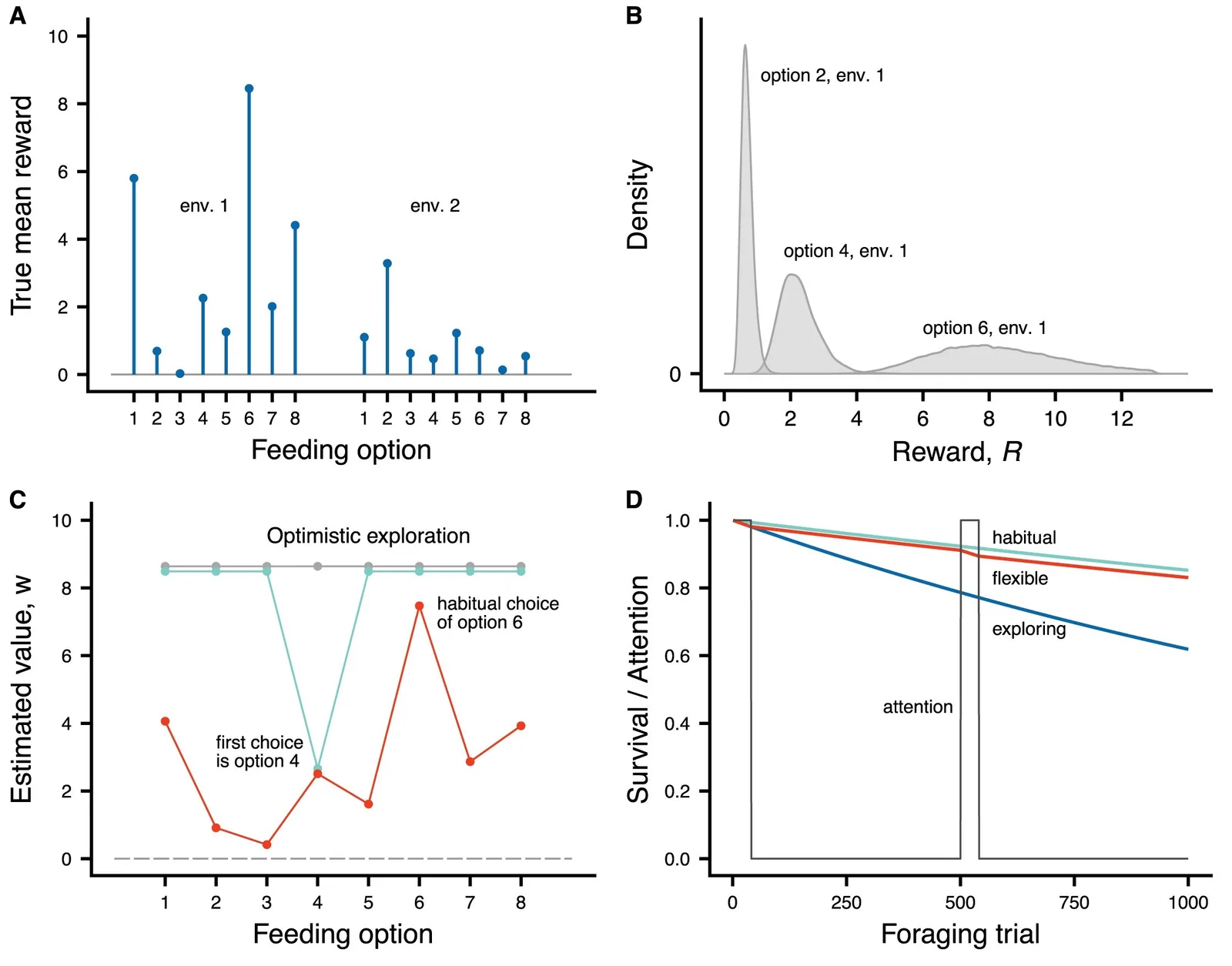
Illustration of model elements. Individuals make and break foraging habits, thereby trading off vigilance and exploration of food sources. As an example, there are two successive environments, each with 500 foraging trials, resulting in a maximal foraging lifetime of 1,000 trials. In each environment, there are eight feeding options, or stimulus types. The options vary in reward distributions between environments. By devoting attention to the exploration of feeding options, individuals can increase their feeding rates, at a cost of decreased vigilance. (A) Mean reward values for the feeding options in two successive environments. Each mean value is independently drawn from a log-normal distribution with a log-scale mean of $\rho _0 = 0$ and log-scale SD of $\sigma _0 = 1$. (B) Examples of the distribution of rewards for three different options, taken from the options available in environment 1. The reward from a given option is log-normally distributed with a given mean and a log-scale SD of $\sigma _R = 0.25$. (C) Illustration of a learning strategy of optimistic exploration. The curves show an individual’s estimate of the values of options (used for action choice) in environment 1 at different points in time. Estimates start high (gray curve). The first choice is option 4, after which estimates are given by the greenish curve. The estimated values after all options are explored and the individual’s behavior is habitual are given by the red curve (curves are shifted slightly to avoid overlap). (D) Mean survival for full habitual foraging (full vigilance; greenish curve; 0.85 at trial 1,000), full attention to exploration (blue curve; 0.62 at trial 1000), and an intermediate case of initially exploring each environment and then becoming habitual (red curve). The gray curve shows a corresponding switching on and off of attention for a flexible strategy.

Our approach is to run individual-based simulations in which foragers have evolving traits, including an initial value estimate ($w_0$), a value learning rate ($\alpha _w$), a choice consistency update rate ($\alpha _h$), and a habit-formation consistency threshold ($\hat{h}$), as detailed in the *Methods* section. Figure 2 shows the evolutionarily stable outcome for a situation as in Figure 1. The regret values in Figure 2A and C are theoretical quantities (deviations of expected reward of actual choice from the expected reward of an optimal choice), and are not observed by foraging individuals but are instead often used to evaluate the performance of exploration–exploitation strategies (Auer et al., 2002; Russo et al., 2018). The red curve in Figure 2A shows that the regret is very close to zero during trials with habitual foraging, while the survival stays close to a maximal full-vigilance value. This illustrates the potential efficiency of the forming and breaking of habits. The example in Figure 2C and D illustrates exploration in an environment where one option (number 3) is most rewarding. After 14 trials of exploration, the individual switches to habitual foraging.

**Figure 2.**
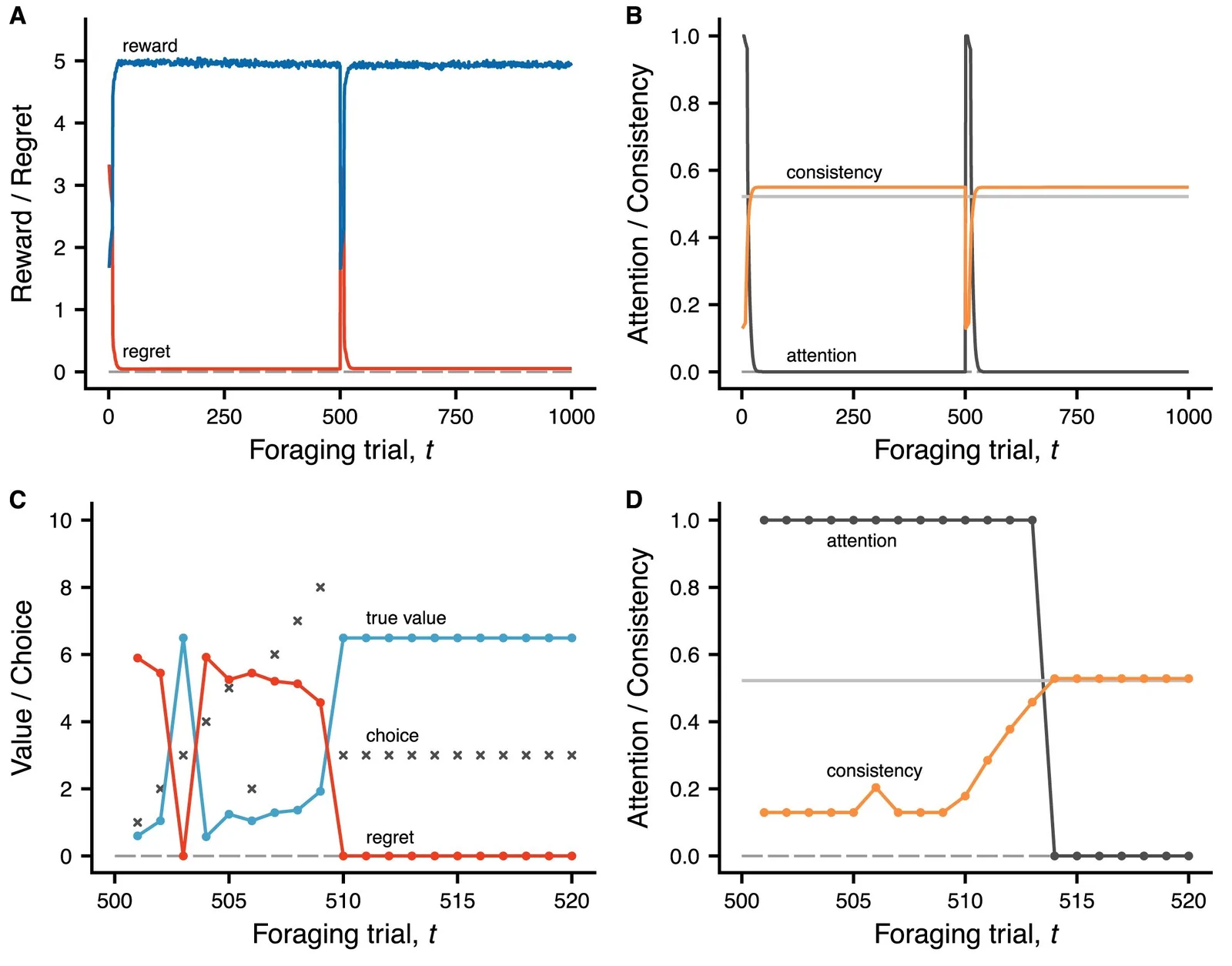
The forming and breaking of habits at an evolutionary equilibrium, for a situation as illustrated in Figure 1. In each of two successive environments, there are eight feeding options, with expected reward values drawn from a log-normal distribution (log-scale mean $\rho _0 = 0$ and SD $\sigma _0 = 1$) and trial-to-trial random reward variation as in Figure 1B ($\sigma _R = 0.25$). At the start of each environment (in trials *t = 0* and *t = 500*), individuals observe a reliable cue, indicating that the environment has changed. There are a total of 4,000 simulated individuals, with traits for optimistic exploration and habit formation at evolutionary equilibrium. Panel A shows the mean over all individuals of the reward in each trial (blue curve), together with the mean regret (red curve). An individual’s regret in a trial measures how much the individual lost by not choosing the option with the maximum expected reward (zero regret indicates an optimal choice). The mean attention to foraging (dark gray) and the mean consistency of choice (orange) are in panel B. Individuals turn off attention to foraging and become habitual when consistency goes above an evolved threshold $\hat{h}$, indicated by a gray horizontal line, after which the update of consistency stops. The consistency measures the degree to which the current choice is the same as the most recent choice. Panels C and D show an example of one individual, for the first 20 trials of the second environment. The successive choices are indicated by dark gray crosses in panel C, together with the corresponding expected (true) values *w* (blue-green) and regret values (red). The average survival over 1,000 trials for the habit-forming individuals is around 0.84, close to the maximal value for full vigilance, and better than the expected survival of 0.62 for full attention to exploration. Trait values: $w_0 = 9.36$, $\alpha _w = 0.90$, $\alpha _h = 0.13$, $\hat{h} = 0.52$.

By modifying the amount of randomness in rewards, we can test the observation from psychology that habits are formed more readily when rewards are more predictable (Buabang et al., 2025; Miller et al., 2019). Our evolutionary model provides support for this idea. As illustrated in Figure 3, the time until habit formation in a new environment is about three times longer when rewards are relatively noisy, compared to a case of little random reward variation around the mean value of options.

**Figure 3.**
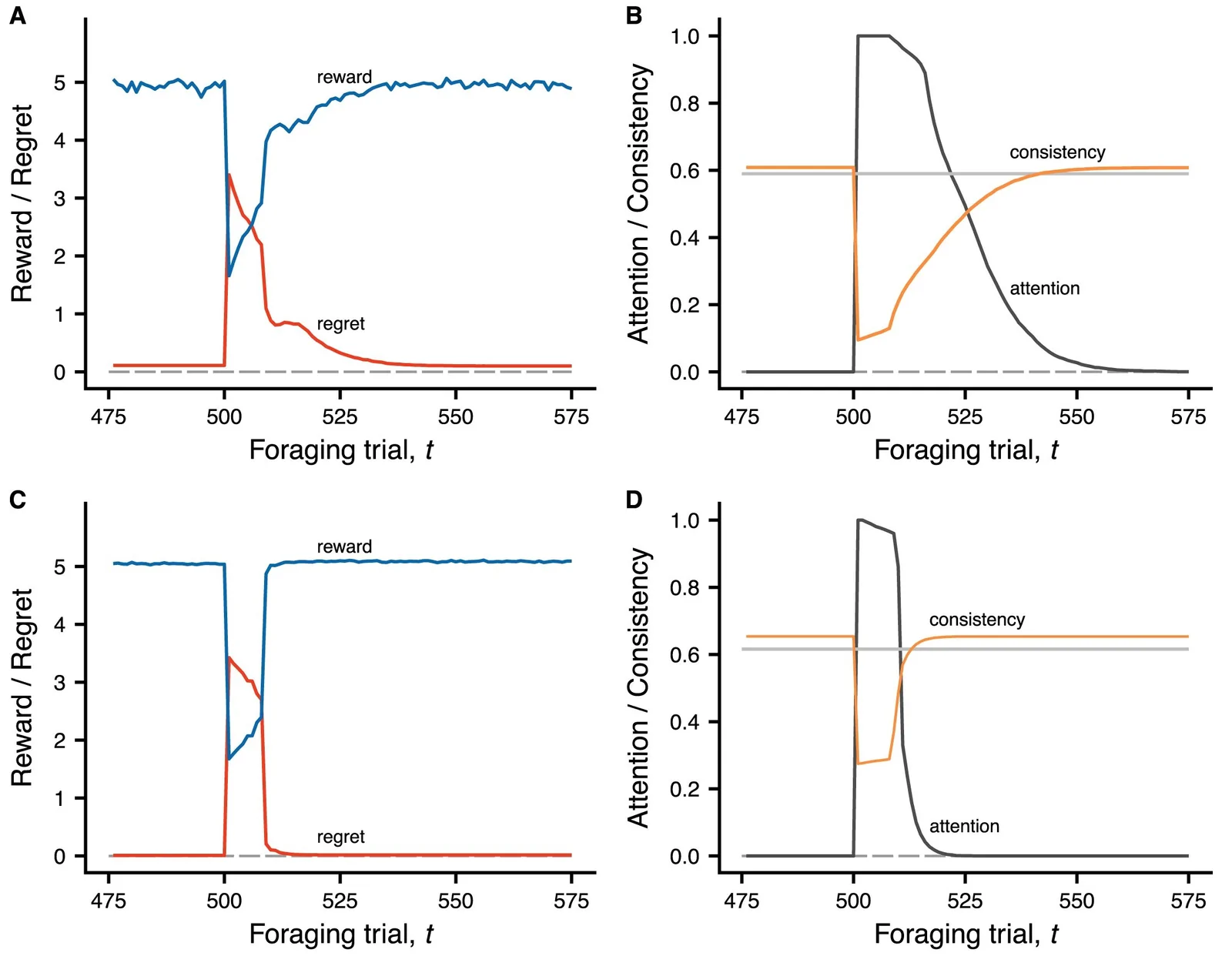
The influence of the predictability of rewards on the speed of habit formation. Illustration of means of reward, regret, attention, and choice consistency for evolutionary equilibria of traits of optimistic exploration and habit formation. The situations are similar to Figure 2, except that the random variation of rewards for a given option is high ($\sigma _R = 0.50$) in panels A and B, and low ($\sigma _R = 0.10$) in panels C and D. The panels show trials around the time of environmental change at *t = 500* trials. With less predictable rewards (A, B) habitual foraging takes longer to develop compared to the case with more predictable rewards (C, D). A factor behind the difference is that the consistency update rate $\alpha _h$ is lower in panels A and B than in panels C and D. At evolutionary equilibrium, the trait values for lower, higher predictability are $w_0 = 5.73, 11.80$, $\alpha _w = 0.66, 0.95$, $\alpha _h = 0.09, 0.27$, $\hat{h} = 0.59, 0.62$.

The evolutionary simulations for Figure 3 illustrate a notable property of the exploration strategy we use here (OE). When observations are noisy, in the sense that random variation ($\sigma _R$) is comparable to (or even larger than) variation among mean rewards of feeding options, the initial estimate of reward ($w_0$) evolves to a smaller value; $w_0 \approx 6$ in Figure 3A and B and $w_0 \approx 12$ in Figure 3C and D. This means that the term “optimistic exploration” is appropriate mainly for observations with relatively little noise. Another thing to note is that the value learning rate $\alpha _w$ evolved to a lower value in the situation with more random variation in rewards ($\alpha _w = 0.66$ vs. $\alpha _w = 0.95$), and this is in agreement with several previous modeling results on the response of learning rates to stochasticity in rewards (e.g., Leimar et al., 2024).

Intuition might suggest that if exploration and learning do not influence predation risk, habit formation should not evolve, but that is not what our analyses show. As an illustration, the situation in Figure 4 is the same as in Figure 3, except that attention to foraging does not influence predation risk. The outcome is that the forming and breaking of habits still evolve, although the time until habit formation in a new environment is twice as long or more in Figure 4 compared to Figure 3. Another difference is that the fitness benefits of habit formation are substantially greater when there are effects of predation risk than without such effects (see supplements on fitness effects of habit formation). The explanation for habit formation in Figure 4 might be that, after a certain amount of sampling of options, and given the finite time horizon, if one is doing well in terms of current rewards it does not pay to continue sampling, because of a risk of sampling poorer options.

**Figure 4.**
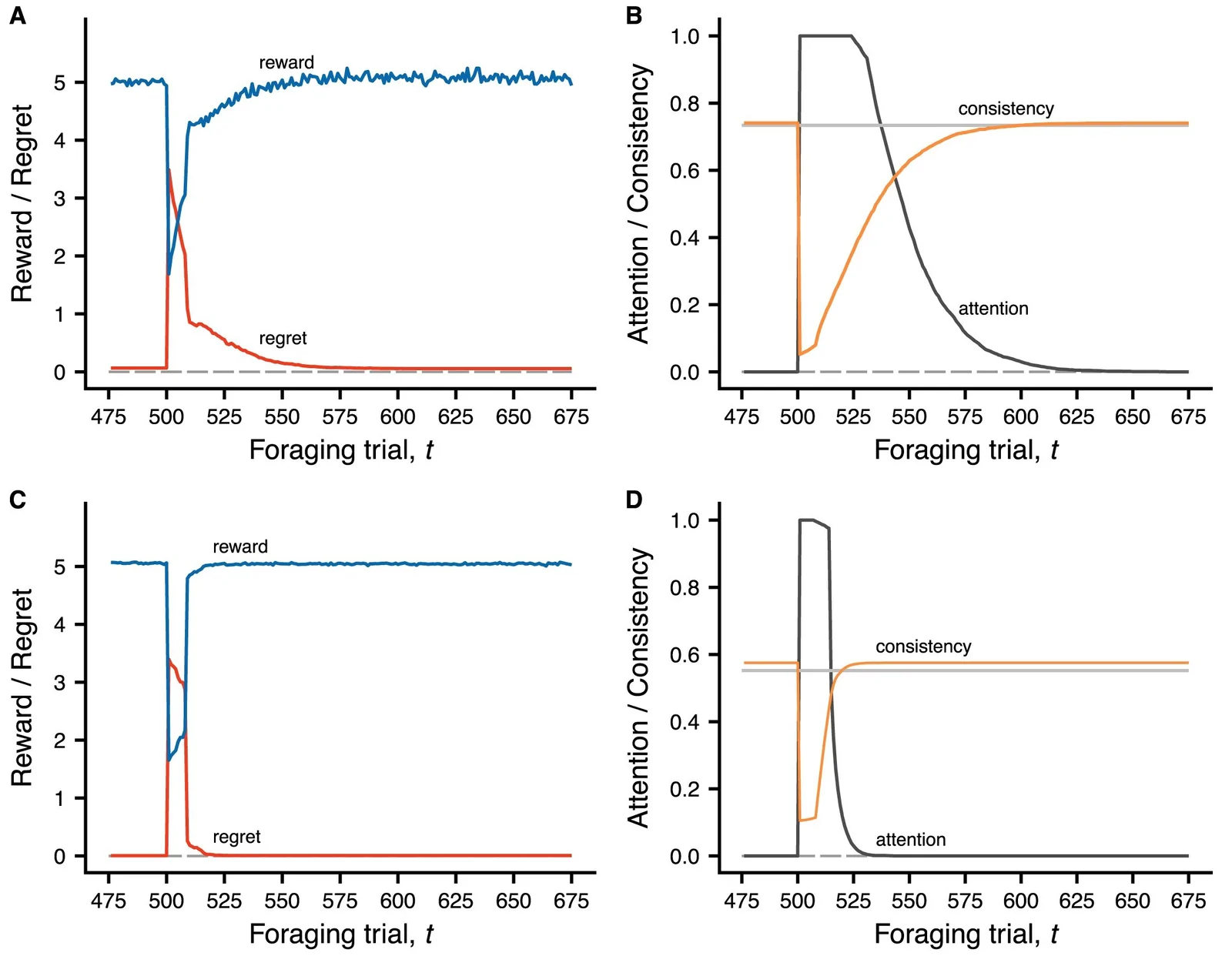
The forming and breaking of habits can also be selectively favored when exploration and learning do not influence predation risk. The reward distributions of options are the same as in Figure 3, but the mortality schedules differ. Here, the attention to exploration and learning does not influence predation risk. The panels show means of reward, regret, attention, and choice consistency for evolutionary equilibria of traits of optimistic exploration and habit formation, for two cases differing in the predictability of rewards. The random variation of rewards for a given option is high ($\sigma _R = 0.50$) in panels A and B, and low ($\sigma _R = 0.10$) in panels C and D. The panels show trials around the time of environmental change at *t = 500* trials. With less predictable rewards (A, B) habitual foraging takes longer to develop compared to the case with more predictable rewards (C, D), and each of these times is longer than is the case in Figure 3 (note that the scales on the x-axes differ between Figures 3 and 4). At evolutionary equilibrium, the trait values for lower, higher predictability are $w_0 = 5.14, 12.74$, $\alpha _w = 0.63, 0.94$, $\alpha _h = 0.05, 0.11$, and $\hat{h} = 0.73, 0.55$.

This raises the question of when habit formation should be selectively disadvantageous. Among the various possibilities, our analyses identify high environmental volatility, in the sense of more rapidly changing environments, in combination with little or no influence of exploration and learning on predation risk, as disfavoring the formation of habits (see Supplementary Figure S1, which also illustrates the effects of less reliable cues of environmental change).

In the supplements, we also show results corresponding to Figures 2, 3, and 4, but using TS instead of OE (Supplementary Figures S2–S4; see also Supplementary Figure S5). Overall, our results support a general conclusion about selection for the forming and breaking of habits: a clear separation among the reward distributions of available feeding options (i.e., predictable rewards), together with readily available habit-breaking indicators of new environments, and with environments staying relatively constant for an appreciable period, act to promote the evolution of habit-mediated exploration–exploitation strategies and promote rapid habit formation in new environments.

The strength of selection on the traits in our model, including the traits involved in habit formation, can vary over the range of values of a trait. There is sometimes quite weak stabilizing selection at trait equilibria, resulting in auto-correlated fluctuations of population mean traits over evolutionary time. In the supplements, we exemplify these fitness effects and illustrate the evolutionary dynamics (Supplementary Figures S6 and S7).

Concerning the breaking of habits, our assumption about cues of environmental change is that they are easy to detect and do not require extra attention. This need not always be the case, and in the supplements we implement an alternative mechanism of habit breaking, where habits can be broken by a sequence of low rewards. Thus, environmental change might cause habitually chosen options to deliver low rewards, so responding to low rewards can be one way of detecting environmental change. Using this alternative habit-breaking mechanism, we find qualitatively similar results (Supplementary Figures S8 and S9) to those for reliable cues of change (Figures 2 and 3), although with noisy rewards, the alternative mechanism is less efficient than reliable cues of change (see Supplementary Figure S9A).

From the various cases that we have analyzed, including those in the supplements, we can draw a few general conclusions about the factors influencing the evolution of formation and breaking of habits. Overall, habit formation will be faster or occur more readily if either the benefits of multitasking are greater or the learning investments required for successful foraging are smaller. An example is when freeing up attention to predator detection results in greater decreases in mortality (Figure 3 vs. Figure 4; Supplementary Figure S1, Supplementary Figure S3 vs. Supplementary Figure S4). Another example is that less noisy reward, for which options can be learnt about more quickly, speeds up habit formation (Figure 3; Supplementary Figures S3 and S9). Yet another example is that high volatility, in the form of frequent environmental changes, in combination with small or no benefits of multitasking, selects against habit formation (Supplementary Figure S1). Finally, clear and unambiguous cues of environmental change are more effective for habit breaking than using estimates of recent rewards, in particular when rewards are noisy (Figure 3 vs. Supplementary Figure S9).

## Discussion

Based on our evolutionary analyses, we propose that the forming and breaking of habits is an important type of behavioral flexibility. The flexibility is advantageous over a range of situations, including when there are wholesale environmental changes. Our evolutionary perspective corroborates current views on habits in psychology (Bouton, 2021, 2024; Buabang et al., 2025). An example of a point of agreement is that habit formation is known to be promoted by periods of environmental stability, and also by the repetition of actions that yield predictable rewards (Buabang et al., 2025; Miller et al., 2019), which is in accordance with our results in Figure 3. We also found that habits form more quickly when the benefits of multitasking are greater (comparing Figures 3 and 4), which is intuitive but not previously studied.

In psychological studies of learning, changing environments are often discussed in terms of volatility of rewards (Nassar et al., 2010), with reversal of rewards perhaps being the most studied (Izquierdo et al., 2017; Miller et al., 2019; Soltani & Izquierdo, 2019), together with the case of continual shifting of the reward values or the probabilities of reward (Behrens et al., 2007; Daw et al., 2006). These forms of volatility correspond to aspects of environments encountered in nature, and could matter for habitual responses to specific stimuli or situations. In our work here, the main focus is on the forming and breaking of habits as a consequence of wholesale environmental changes.

Different meanings have been given to the term “behavioral flexibility” (Audet & Lefebvre, 2017; Lea et al., 2020; Uddin, 2021), with different experimental measures or tests of such flexibility. Reversal learning is perhaps the most commonly used test, and it is in this general sense we are suggesting that the forming and breaking of habits is a form of behavioral flexibility. The specific habit-forming and -breaking mechanisms assumed in our models are likely to be simplifications of real mechanisms. Thus, habits may form gradually (Buabang et al., 2025; Miller et al., 2019) rather than through a threshold mechanism. In our main model, the breaking of habits depends on specific cues, in accordance with work on the effects of context changes (Bouton, 2021, 2024; Buabang et al., 2025). As an alternative possibility, inspired by work in neuroscience on detecting and reacting to unexpected changes in the world (Cohen et al., 2007; Dayan & Yu, 2006; Yu & Dayan, 2005), we found that a lowering of the rate of rewards below a threshold can evolve to be a habit-breaking mechanism.

Habit formation in our model is a process of first exploring options and then switching to habitual foraging. This is qualitatively similar to a strategy of the so-called “satisficing,” as developed in the study of bounded rationality (Gigerenzer & Gaissmaier, 2011; Gigerenzer & Goldstein, 1996; Simon, 1956, 1976). In theories of economic decision-making, a satisficing strategy searches through alternatives until a threshold for acceptability is met, at which time searching stops. In making this comparison, one should note that evolutionary equilibria in our model represent optimal collection of rewards under the constraints of predation risk and a limited time period, where optimality is over the space of strategies given by our trait space (Fawcett et al., 2013). This differs from the current usage of the term satisficing in theories of decision-making: optimization under constraints is regarded as distinct from bounded rationality (Gigerenzer, 2001; Selten, 2001). It then seems that our analysis of the forming and breaking of habits is closely related to but distinct from ideas of bounded rationality in economics. In any case, habit formation is likely to play an important role in human decision-making.

There is a wide field of investigation into mechanisms of learning. The switching on and off of learning in our model can be seen as a version of the much-studied idea that learning rates should respond to environmental characteristics, for instance with higher learning rates in more volatile environments (Dayan et al., 2000; Gershman, 2015; Leimar et al., 2024; Piray & Daw, 2021). Further, the concept of attention has long been used to develop ideas about learning (Lovejoy, 1965, 1966; Mackintosh, 1962, 1969, 1975; Pearce & Hall, 1980). Our model assumption of a simple switching on and off of attention to exploration of feeding options should be seen as a first-step simplification of possibly complex dynamics, involving gradation and shifts between different reward-predicting stimulus dimensions. Even so, there is an empirically well-supported tradition in psychology and neuroscience of the so-called dual-process models, with essentially discrete modes of behavior (Buabang et al., 2025; Daw et al., 2005; Dolan & Dayan, 2013; Miller et al., 2019, 2022; Moskovitz et al., 2024). The exploratory and habitual behaviors in our model are meant to represent such modes.

In addition to these models, there could be other ways of being efficient in combining foraging and vigilance. One example is a model where individuals can commit to foraging over a period of several trials, while at the same time being sensitive to indirect, predator-related cues that can interrupt the foraging (Lloyd & Dayan, 2018). Individuals can then forage while also maintaining a certain vigilance, by being sensitive to cues, such as rustling sounds, indicating the presence of a predator. This need not be related to habit formation, but it is also possible that habitual foraging allows an individual to direct more attention to a range of cues that can give information about predation risk.

In general, exploration, foraging, and vigilance are tasks with several demands on mental capacities. For instance, exploration in nature could involve paying attention to and remembering locations and surroundings of different feeding options, in this way showing complexities beyond responding to a multi-armed bandit.

The essential role played by habitual foraging in our model is to enhance multitasking, through improved vigilance with only minor changes in foraging success. The combination of foraging and vigilance is sometimes studied in psychology (Chebolu et al., 2022; Lloyd & Dayan, 2018) and, as mentioned, has long been of interest in behavioral ecology (Houston et al., 1993; Milinski & Heller, 1978). This interest includes the role of attention in detecting predators (Dall et al., 2001; Dukas, 2002, 2004) and the importance of vigilance for survival (Ellsworth et al., 2024). There are experimental approaches to measuring variation in vigilance, for instance comparing the time until detection of a (model) predator between different kinds of aggressive behavior in prey animals (Jakobsson et al., 1995), or a similar comparison for fish prey surrounded by either familiar or unfamiliar conspecifics (Griffiths et al., 2004). Such approaches, involving measuring the reaction time to the presentation of a model predator, could serve to diagnose whether a foraging habit has been formed. This would then be an addition to current experimental methods of diagnosing habitual behavior in psychological research (Bouton, 2021, 2024; Buabang et al., 2025).

A characteristic property of habits is of course the tendency to use a particular action or sequence of actions in a given situation. This could be used to diagnose and thus establish a timescale for the shift from exploration to exploitation of a foraging individual. For habit formation to be advantageous in nature, this timescale should be shorter than the timescale of significant environmental change. In addition to seasonal changes and new locations, one needs to also take into account other changes, such as those deriving from foraging itself. The latter will be particularly relevant for group-living animals with frequency dependence in the rewards of foraging.

In addition to foraging, there are other ecologically significant activities that sometimes need to be combined with antipredatory vigilance, for instance social behavior, like aggression, caring for offspring, and attracting mates. The formation of habits for these activities might then be advantageous. Further, if developmental plasticity from early learning leads to variation in consistent behavioral traits such as boldness and aggression (Frost et al., 2006), habit formation might contribute to animal personality variation. A general conclusion is then that further study of habits should be worthwhile for evolutionary and behavioral ecology, including through modeling, field observation, and ecologically motivated experiments.

## Supplementary Material

qrag024_Supplemental_File

## Code and code availability

C++ source code for the individual-based simulations is available at Zenodo: DOI: https://doi.org/10.5281/zenodo.20569011

## Appendix A

In many of his works, Charles Darwin was concerned with the nature of habits, in particular with the possible significance of habits for adaptation and evolutionary change (Gordillo-García, 2016; Portera & Mandrioli, 2021). Darwin’s early thinking went along the lines of the inheritance of acquired characters, whereby habits that are acquired within an individual’s lifetime and are helpful for reproduction and survival could be transformed into inherited traits. Still, already in the first edition of the *Origin of Species*, Darwin rejected such Lamarckian ideas, while continuing to argue that there were instances where acquired habits somehow contributed to the evolution of instincts (e.g., Darwin, 1859, pp. 207–209).

From a current perspective, these instances might be interpreted as the so-called “genes as followers” or “plasticity first” evolution (West-Eberhard, 2003, 2005), with a possible example being learnt foraging habits contributing to the diversification of Darwin’s finches in the Galápagos Islands. In addition to this issue of the evolutionary origins of innate behavior, which is fundamental but is not the topic of our work here, Darwin also noted that habits allow complex behaviors to be performed seemingly automatically, which is a property of habits we study here.

## Appendix B

Here, we give brief explanations of some terminology we use. The terminology is standard in fields like experimental psychology or machine learning, but might not be familiar to evolutionary biologists. In psychological sciences, a distinction is made between goal-directed actions, that are made with the outcome of the action in mind, and habitual actions, which are essentially automatic responses to stimuli. If obtaining a reward is an outcome of an action in response to a stimulus, there can be learning to perform this as a goal-directed action. With sufficient repetition, the action can become a habitual response to the stimulus. The exploration–exploitation trade-off is a basic concept in decision theory, and amounts to the question of how a decision-maker should balance obtaining information about the consequences of making different choices and efficiently making use of the information already obtained. The term multi-armed bandit is inspired by the one-armed bandits, or slot machines, used for gambling, and represents a decision problem with several choices with different rewards, or probabilities of reward. Action-value learning is a simple form of reinforcement learning where a decision-maker forms estimates of the values of taking different actions and then uses these estimated values to make choices, for instance using the *ε*-greedy method.
