# Supplementary material for: Evolution of behavioral flexibility and the forming and breaking of habits
Source: Evol Lett. 2026 Jun 19;10(5):463–74. doi: 10.1093/evlett/qrag024 (PMC13629241; doi:10.1093/evlett/qrag024)
Supplement: Supplementary file 1 [file qrag024_supplemental_file.pdf]

Supplements to: Evolution of behavioral flexibility and the forming and breaking of habits

Olof Leimar[1, *], Sasha R. X. Dall[2], Peter Hammerstein[3], Alasdair I. Houston[4], Bram Kuijper[5], and John M. McNamara[6]

[1]Department of Zoology, Stockholm University, 106 91 Stockholm, Sweden; olof.leimar@zoologi.su.se

[2]Centre for Ecology and Conservation, University of Exeter, Penryn TR10 9FE, UK; S.R.X.Dall@exeter.ac.uk

[3]Institute for Theoretical Biology, Humboldt University Berlin, 10115 Berlin, Germany; p.hammerstein@biologie.hu-berlin.de

[4]School of Biological Sciences, University of Bristol, Bristol, BS8 1TQ, UK; A.I.Houston@bristol.ac.uk

[5]Centre for Ecology and Conservation, University of Exeter, Penryn TR10 9FE, UK; A.L.W.Kuijper@exeter.ac.uk

[6]School of Mathematics, University of Bristol, Bristol, BS8 1UG, UK; john.mcnamara@bristol.ac.uk

[*]Corresponding author; e-mail: olof.leimar@zoologi.su.se

Supplementary information

Cues of environmental change

We assume that, on the first trial of a potentially new environment, an individual observes an environmental cue z_{e}, indicating the probability that the environment has changed. For the results in the main text, we use 'reliable cues', i.e. cues that indicate a probability close to one. We assume that z_{e} is normally distributed with mean μ_{e} and standard deviation σ_{e}, and a relation between the cue value and the probability of environmental change as in logistic regression, such that

$$p_{\mathrm{e}} = \frac{1}{1+\exp(-z_{\mathrm{e}})} \tag{S1}$$

is the probability of a change. Individuals use a threshold $\hat{e}$ to determine if they should restart exploration, either OE or TS, and break any current habit, resetting if

$$p_{\mathrm{e}} > \hat{e}. \tag{S2}$$

The parameters $\hat{h}$ and $\hat{e}$, influencing the forming and breaking of habits, are implemented as genetically determined traits in our evolutionary simulations.

For the results in the main text, we use the parameter values $\mu_e = 8.0$, $\sigma_e = 1$, in which case the cues are highly reliable indicators of change.

Illustration of effects of high environmental volatility

In order to identify situations where habit formation does not evolve, here we examine cases where there can be many environmental changes during an individual's lifetime (up to 50 environments), with each environment potentially having a short duration of only 20 trials. If each such environment is drastically different, this might be an unrealistically high volatility, so we also examine cases where successive environments are fairly similar, in the sense of small values of the parameter σ_0. We combine a range of cases with different values of σ_0, with ρ_0 adjusted to keep the overall mean rewards constant, with cases of different influence of attention to foraging on predation risk, having either $\mu_1 = 0.00032$ or $\mu_1 = 0.0$ in equation (2) in the main text.

Our model assumes that individuals can observe a cue that can indicate a change of environment, or context. In our examples in the main text, these cues have been highly accurate in predicting change, but it is also of interest to investigate the effect of cues of lower accuracy. For high volatility, it could be realistic that cues indicating change

are of intermediate reliability. We implement this by having a quantitative cue and letting the cue strength indicate the probability that the environment has changed, as in equation (S1). If this probability is small and the different environments are relatively similar, it might be best for habitual foragers to ignore the cue, thus avoiding predation costs of resetting attention to exploration.

These possible effects, together with the influence of exploration and learning on predation risk (parameter μ_1), and the magnitude of change between environments (parameter σ_0), are illustrated in Fig. S1. A main result is that habit formation is not selected for if there is a combination of high environmental volatility and no influence of exploration and learning on predation risk.

Thompson sampling (TS)

Thompson sampling (Thompson 1933) is a Bayesian statistical approach with near optimal or optimal exploration-exploitation performance in certain situations (Russo et al. 2018), but it is not known if it is approximated by cognitive mechanisms in real animals. We use this form of exploration for some of the results here, to give a comparison to the OE approach in the main text.

As mentioned, we assume that the different feeding options have log-normal reward distributions. So if option k, $k = 1, \ldots, K$, is chosen in trial t, we assume that the reward $R_k(t)$ is drawn from a log-normal distribution with parameters ρ_k and σ_R, i.e., $y_k(t) = \log(R_k(t))$ is normal with mean ρ_k and standard deviation σ_R. For convenience we sometimes use the precision $\tau_R = 1/\sigma_R^2$ as parameter instead of σ_R. In order to have analytic expressions for the posterior distributions, we further assume as prior that the parameters ρ_k of the options are (independently) drawn from a normal distribution with parameters ρ_0 and $\tau_0 = 1/\sigma_0^2$, just as for our analyses of OE. Having observed y_{ki}, $i = 1, \ldots, n_k$ or, as a sufficient summary of observations, $\bar{y}_k = 1/n_k \times \sum_{i=1}^{n_k} y_{ki}$, the posterior distribution for ρ_k,

$$\mathrm{p}\big(\rho_k \,|\, y_{k1}, \ldots, y_{kn_k}\big),$$

is then normal with mean

$$\hat{\rho}_{kn_k} = \frac{\tau_0 \rho_0 + n_k \tau_R \bar{y}_k}{\tau_0 + n_k \tau_R} \tag{S3}$$

and precision

$$\hat{\tau}_{kn_k} = \tau_0 + n_k \tau_R, \tag{S4}$$

with $\hat{\sigma}^2_{kn_k} = 1/\hat{\tau}_{kn_k}$. This posterior is then used for the sampling of parameters.

The sampling and action choice procedure

The Thompson sampling procedure is, for each k, to 'draw' parameters $\hat{y}_k$ from the normal distributions with parameters from equations (S3, S4), and then to choose the option with the largest posterior mean reward, i.e., with the largest value of

$$\hat{R}_{kn_k} = \exp\bigl(\hat{y}_{kn_k} + \sigma^2/2\bigr), \tag{S5}$$

which is the same as choosing the option with the largest value of $\hat{y}_{kn_k}$.

Comparing optimistic exploration with Thompson sampling

Figures S2, S3, and S4 show results that correspond to Figs. 2, 3, and 4 in the main text, with OE replaced by TS as the exploration strategy. Comparing Figs. 2 and 3 with S2 and S3, one sees that in these situations there is qualitative similarity between OE and TS in how habits are formed, the main difference being that the period of exploration before habit formation is longer for TS than for OE. A likely explanation for this is that OE is initially faster than TS in picking more rewarding options, whereas in the long run TS is more thorough in its exploration.

There is a qualitative difference when comparing Figs. 4 and S4, where exploration does not influence predation risk. For these environments, OE leads to the evolution of habit formation, whereas habit formation is selected against with TS. In terms of rewards collected there is, however, not a big difference, with the overall mean regrets being small and of similar magnitudes for OE and TS.

With less variation in expected rewards between options and more stochastic variation in rewards, there is again qualitative similarity between OE and TS when there is no predation risk, in that there is selection for habit formation both for OE and TS, as is illustrated in Fig. S5.

Fitness effects of habit formation

In our model, an individual's lifetime reproductive success (LRS) is proportional to the lifetime sum of rewards obtained by the individual, up until the individual dies

from predation or until the end of the maximum life span. Comparing the mean of this measure, for the examples in Figs. 3 and 4 in the main text, with simulations where individuals are constrained not to form habits (not shown in figures), we find that there is reduction of LRS from preventing habit formation of around 13% in Fig. 3, but only around 0.6% in Fig. 4. This illustrates that the fitness benefits of habit formation can be substantially greater when there are effects of attention to exploration on predation risk, compared to when there there are no such effects. The fitness effects come from a combination of survival and foraging efficiency. Concerning foraging efficiency, the small regret values during habitual foraging seen in Figs. 2 and 3 imply that there is little loss of foraging efficiency. For the situation in Fig. 3A,B, where regret values are slightly larger in the beginning of a new environment, the loss in total rewards collected over all the trials, compared to a situation where habit formation is prevented, is only around 1% (considering only individuals that survived over the entire 1000 trials).

This stronger selection for habit formation if habitual foraging reduces predation risk is further illustrated in Fig. S6. Starting evolutionary simulations from a point of no habit formation, with predation risk there is fairly rapid evolution of habit formation (Fig. S6). Also, as illustrated by the variation in the population average thresholds $\hat{h}$ (Fig. S6A,C), with at least some habit formation, stabilizing selection towards an evolutionary equilibrium of $\hat{h}$ is quite weak, in particular for OE. The strength of stabilizing selection at the equilibria for different situations is further illustrated in Fig. S7.

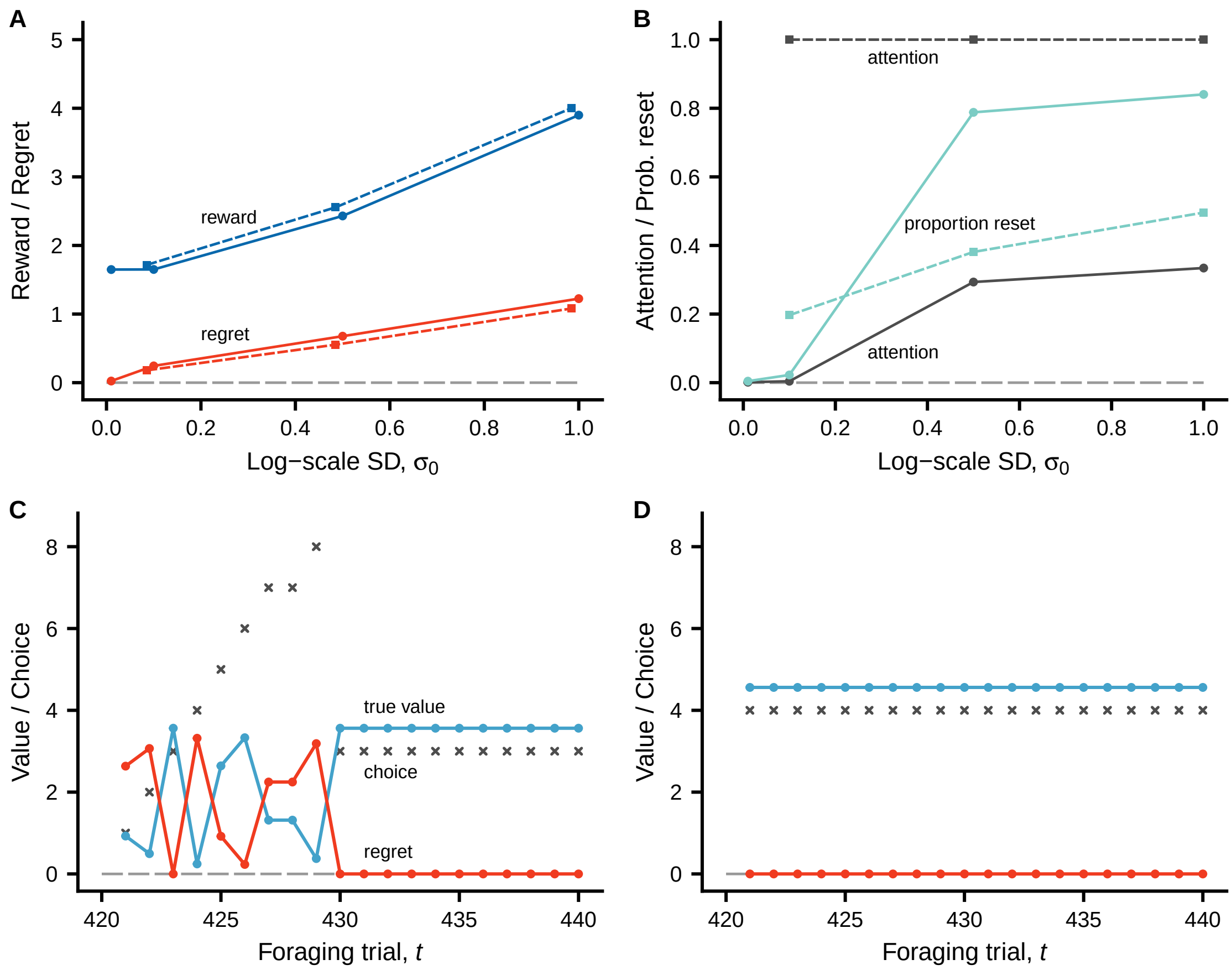

Figure S1: Illustration of the overall means over all individuals and trials (where the individual is alive) of reward, regret, attention, and the probability that an individual resets exploration in response to a cue, for seven cases with different parameter values ρ_0, σ_0 and μ_1. All cases have parameter values $\sigma_R = 0.5$, $\mu_e = 0$, and $\sigma_e = 1$. The values of σ_0 are on the x-axes of panels **(A)** and **(B)**, and solid/round vs. dashed/square symbols indicate $\mu_1 = 0.00032$ vs. $\mu_1 = 0.0$. Note in particular that for $\mu_1 = 0.0$ in panel **(B)**, the evolutionary equilibrium is full attention to exploration and learning, and thus no habit formation, whereas there is forming and breaking of habits for $\mu_1 = 0.00032$. Panel **(C)** illustrates a simulated individual from the $\sigma_0 = 1$, $\mu_1 = 0.00032$ case initiating habitual foraging during a brief span of a new environment lasting 20 trials. Panel **(D)** illustrates a simulated individual from the $\sigma_0 = 1$, $\mu_1 = 0.0$ case correctly avoiding responding to a cue of change.

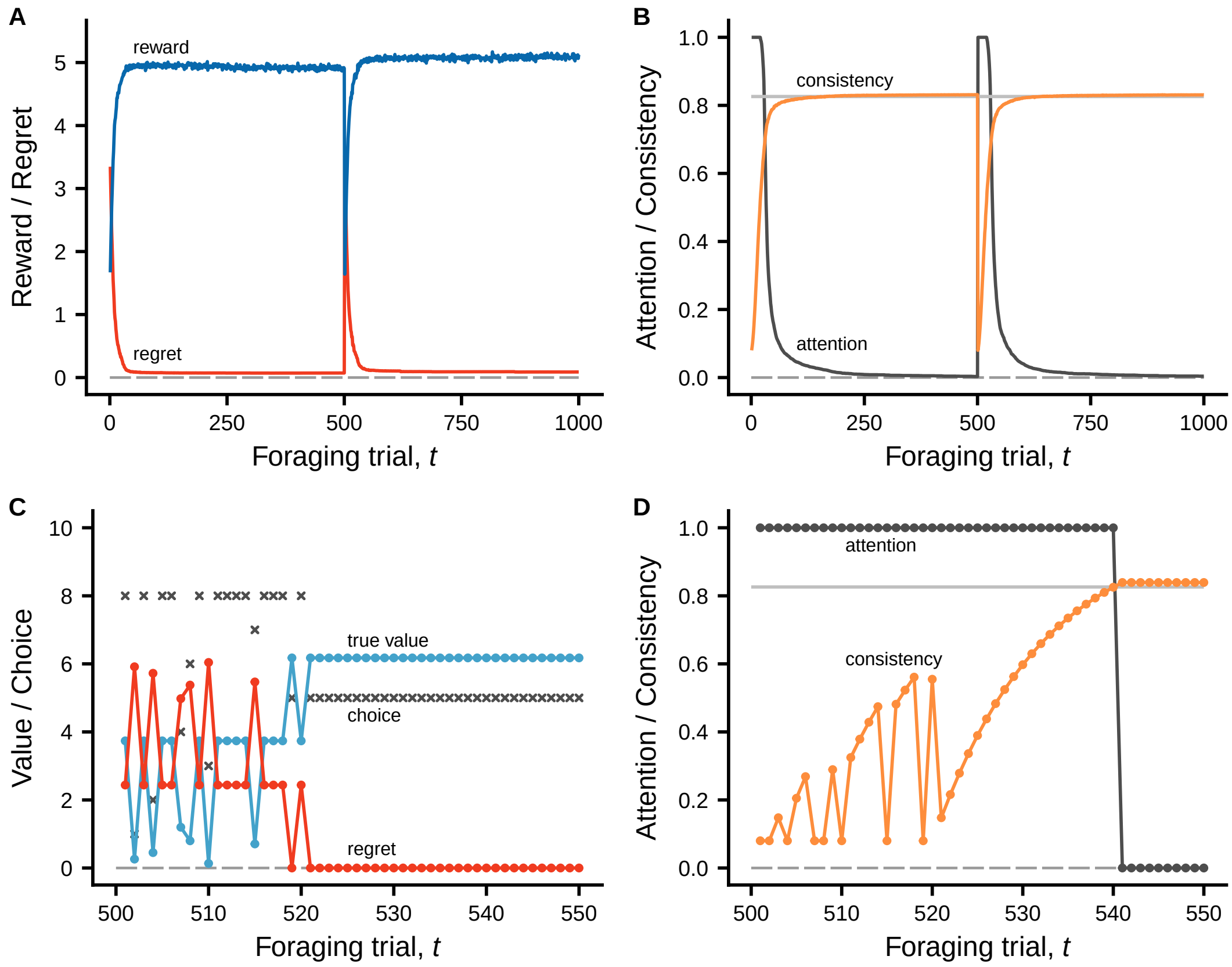

Figure S2: The forming and breaking of habits at an evolutionary equilibrium, corresponding to Fig. 2, but with TS instead of OE. In each of two successive environments, there are eight feeding options, with expected reward values drawn from a log-normal distribution (log-scale mean $\rho_0 = 0$ and SD $\sigma_0 = 1$) and trial-to-trial random reward variation as in Fig. 1B ($\sigma_R = 0.25$). At the start of each environment, individuals observe a cue, indicating that the environment has changed. There are a total of 4000 simulated individuals, with traits for habit formation at evolutionary equilibrium. Panel **(A)** shows the mean over all individuals of the reward in each trial (blue curve), together with the mean regret (red curve). The mean attention to foraging (dark gray) and the mean consistency of choice (orange) are in panel **(B)**. Individuals turn off attention to foraging and become habitual when consistency goes above an evolved threshold $\hat{h}$, indicated by a gray horizontal line, after which the update of consistency stops. The consistency measures the degree to which the current choice is the same as the most recent choice. Panels **(C)** and **(D)** show an example of one individual, for the first 50 trials of the second environment. The successive choices are indicated by dark gray crosses in **(C)**, together with the corresponding expected (true) values w (blue-green) and regret values (red). The average survival over 1000 trials for the habit forming individuals is around 0.83, slightly less than in Fig. 2, but better than the expected survival of 0.62 for full attention to exploration. Trait values: $\alpha_h = 0.08$, $\hat{h} = 0.83$.

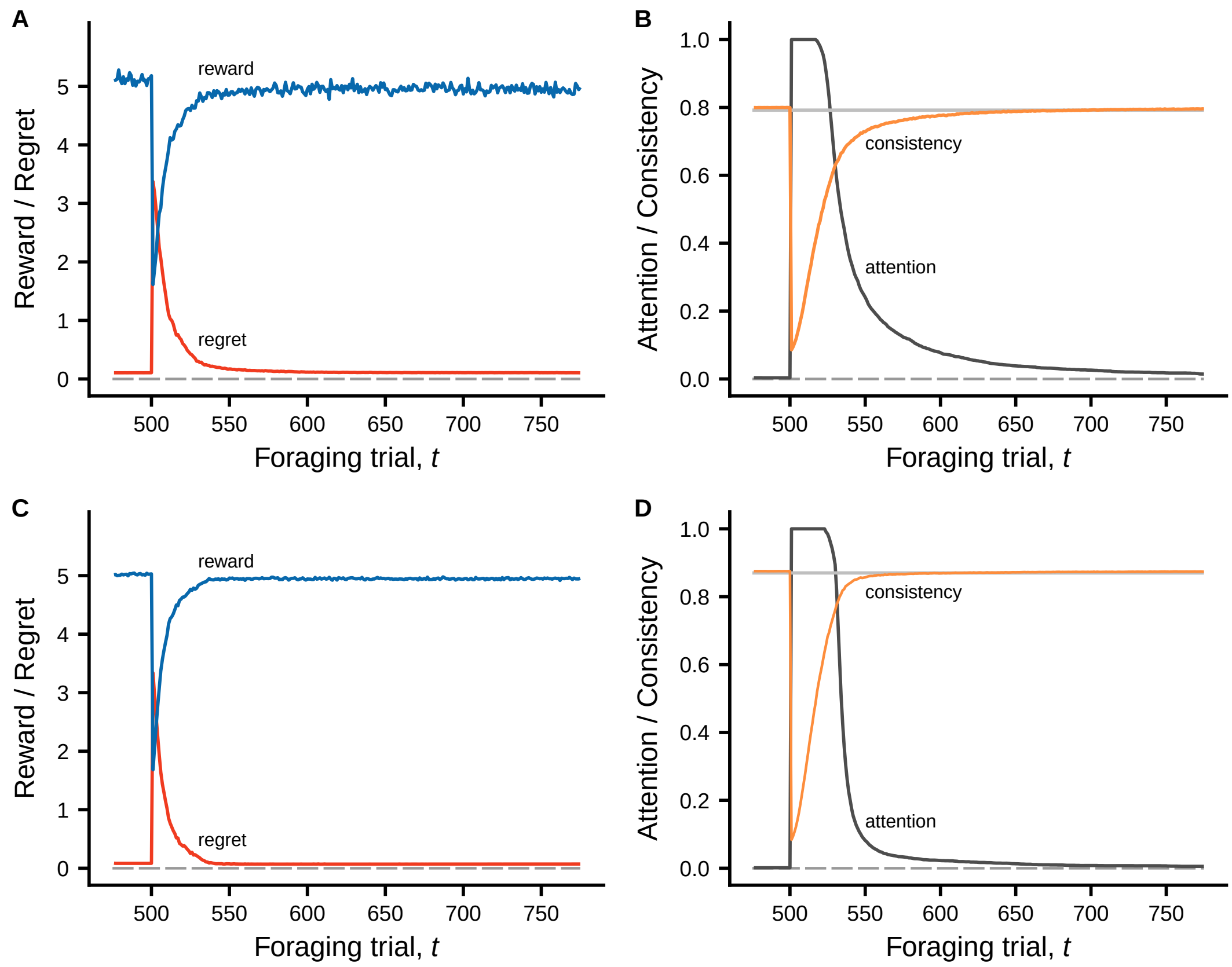

Figure S3: The influence of the predictability of rewards on the speed of habit formation, corresponding to Fig. 3, but with TS instead of OE. Illustration of means of reward, regret, attention, and choice consistency for evolutionary equilibria of traits of habit formation. The situations are similar to Fig. S2, except that the random variation of rewards for a given option is high ($\sigma_R = 0.50$) in panels **(A)** and **(B)**, and low ($\sigma_R = 0.10$) in panels **(C)** and **(D)**. The panels show trials around the time of environmental change at $t = 500$ trials. With less predictable rewards **(A, B)** habitual foraging takes longer to develop compared to the case with more predictable rewards **(C, D)**. A factor behind the difference appears to be that TS entails less repetition of choice in **(A, B)** than in **(C, D)**, resulting in a slower buildup of consistency of choice $h_k(t)$. At evolutionary equilibrium, the trait values for lower, higher predictability are $\alpha_h = 0.086, 0.084$, $\hat{h} = 0.79, 0.87$.

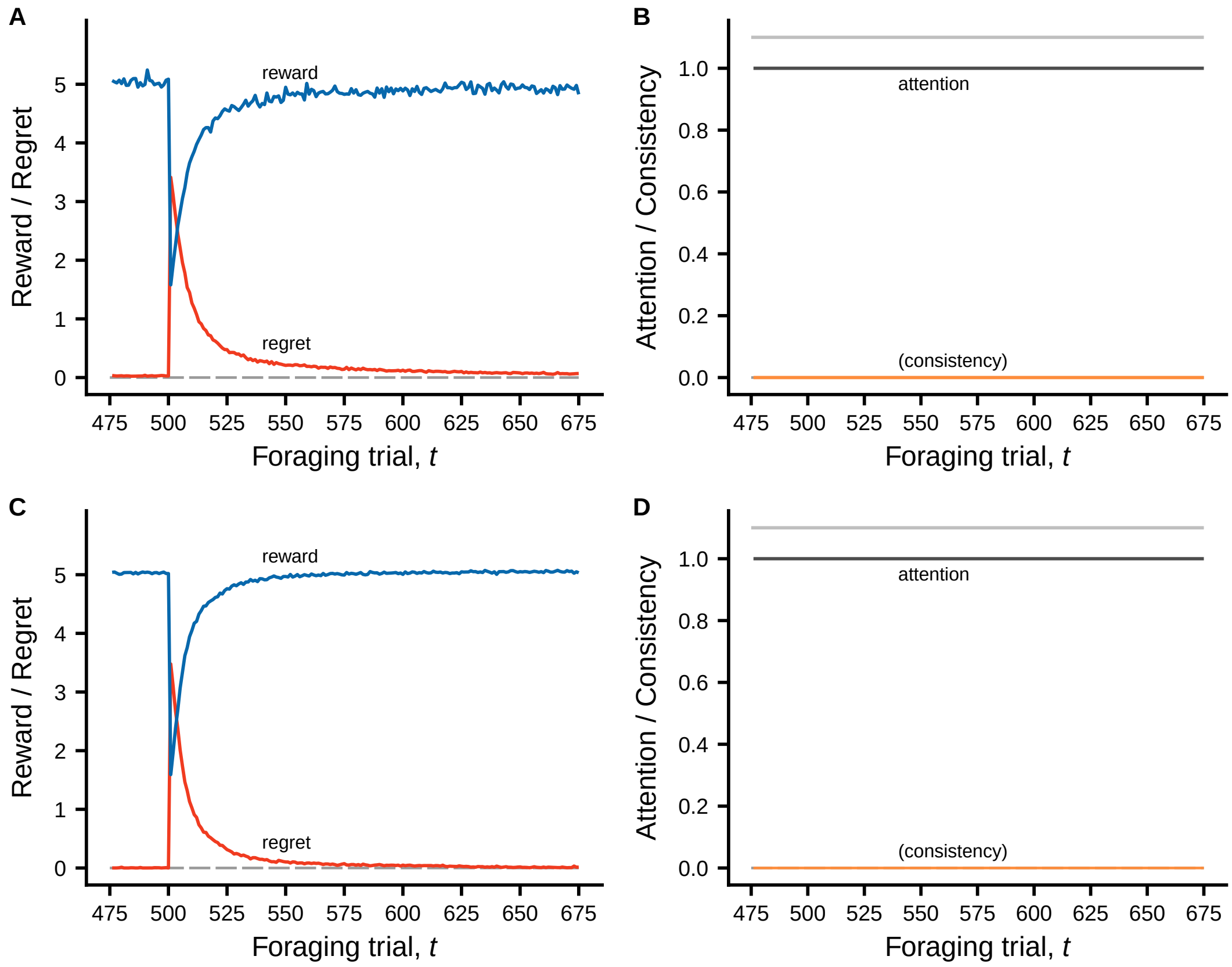

Figure S4: For TS and with environmental volatility/stochasticity as here, the forming and breaking of habits is selected against when exploration and learning do not influence predation risk. For our model, habit formation can be prevented if the habit-formation consistency threshold $\hat{h}$ evolves to a value greater than one, which happens in these cases. The reward distributions of options are the same as in Fig. S3, but here the attention to exploration and learning does not influence predation risk. The panels show means of reward, regret, and attention for TS. Because the trait $\hat{h}$ evolved to $\hat{h} > 1$ in both these cases, choice consistency is irrelevant for habit formation and attention. The random variation of rewards for a given option is high ($\sigma_R = 0.50$) in panels **(A)** and **(B)**, and low ($\sigma_R = 0.10$) in panels **(C)** and **(D)**. The panels show trials around the time of environmental change at $t = 500$ trials.

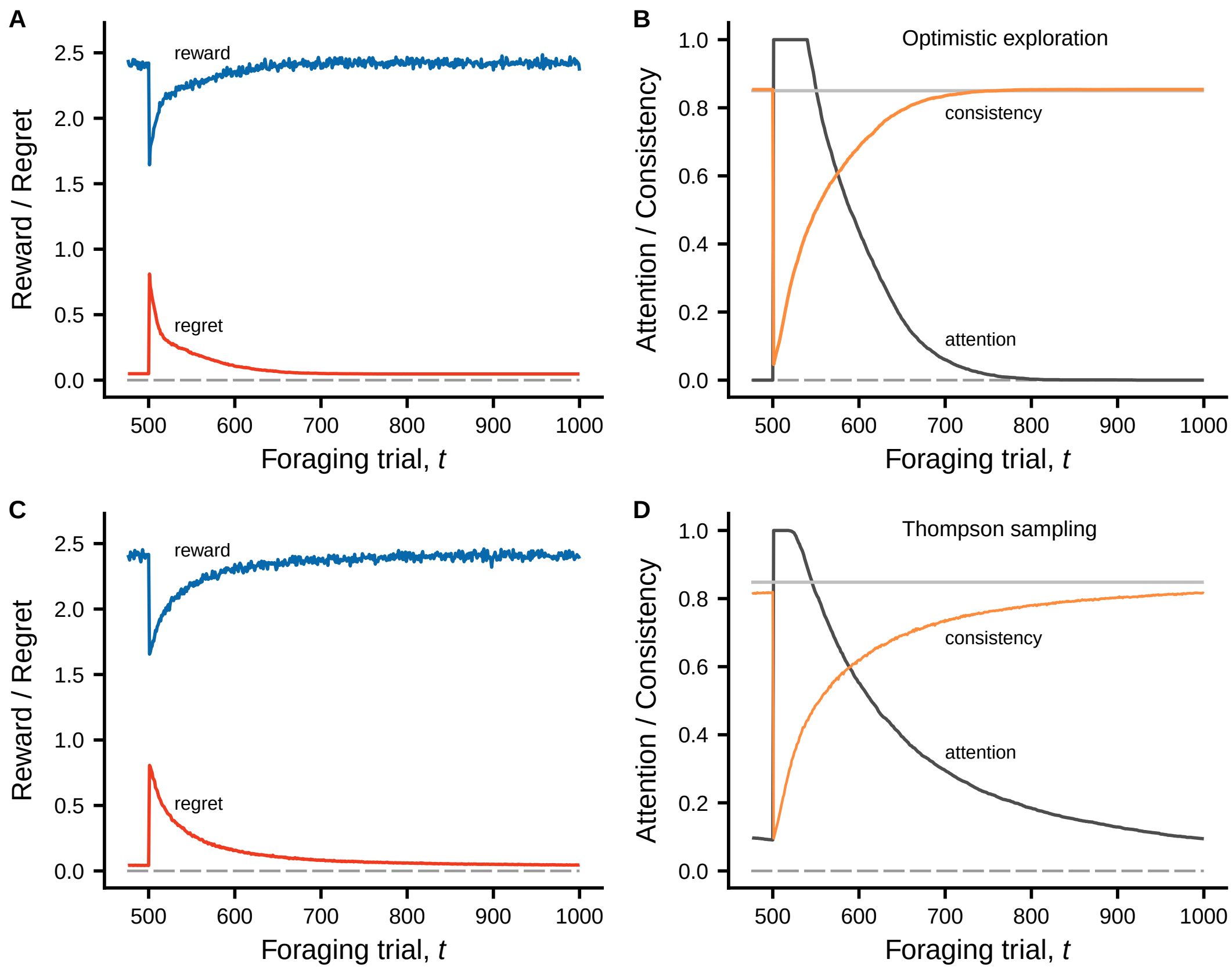

Figure S5: Comparison of exploration and habit formation between OE, in **(A)** and **(B)**, and TS, in **(C)** and **(D)**, for a case with fairly little variation between options in expected rewards, but considerable reward stochasticity, resulting in low predictability. The parameters of the reward distributions are $\rho_0 = 0.455$, $\sigma_0 = 0.30$, and $\sigma_R = 0.5$. There are two successive environments, each of 500 trials, without any mortality ($\mu_0 = \mu_1 = 0$), so there is no predation advantage of habitual foraging. Panels **(A, C)** show mean reward and regret, and **(B, D)** show attention to exploration and consistency of choice, for 4000 simulated individuals, over the trials in the second environment. Times until habit formation are longer than for the examples in the main text and in Figs. S2, S3, S4. For TS the time until habit formation is very variable among individuals, with a fraction of the individuals continuing to explore over the entire duration of the environment, as seen in **(D)**. The trait values are at an evolutionary equilibrium, with $w_0 = 2.10$, $\alpha_w = 0.16$, $\alpha_h = 0.05$, $\hat{h} = 0.85$, $\hat{e} = 0.48$ for OE and $\alpha_h = 0.10$, $\hat{h} = 0.85$, $\hat{e} = 0.40$ for TS.

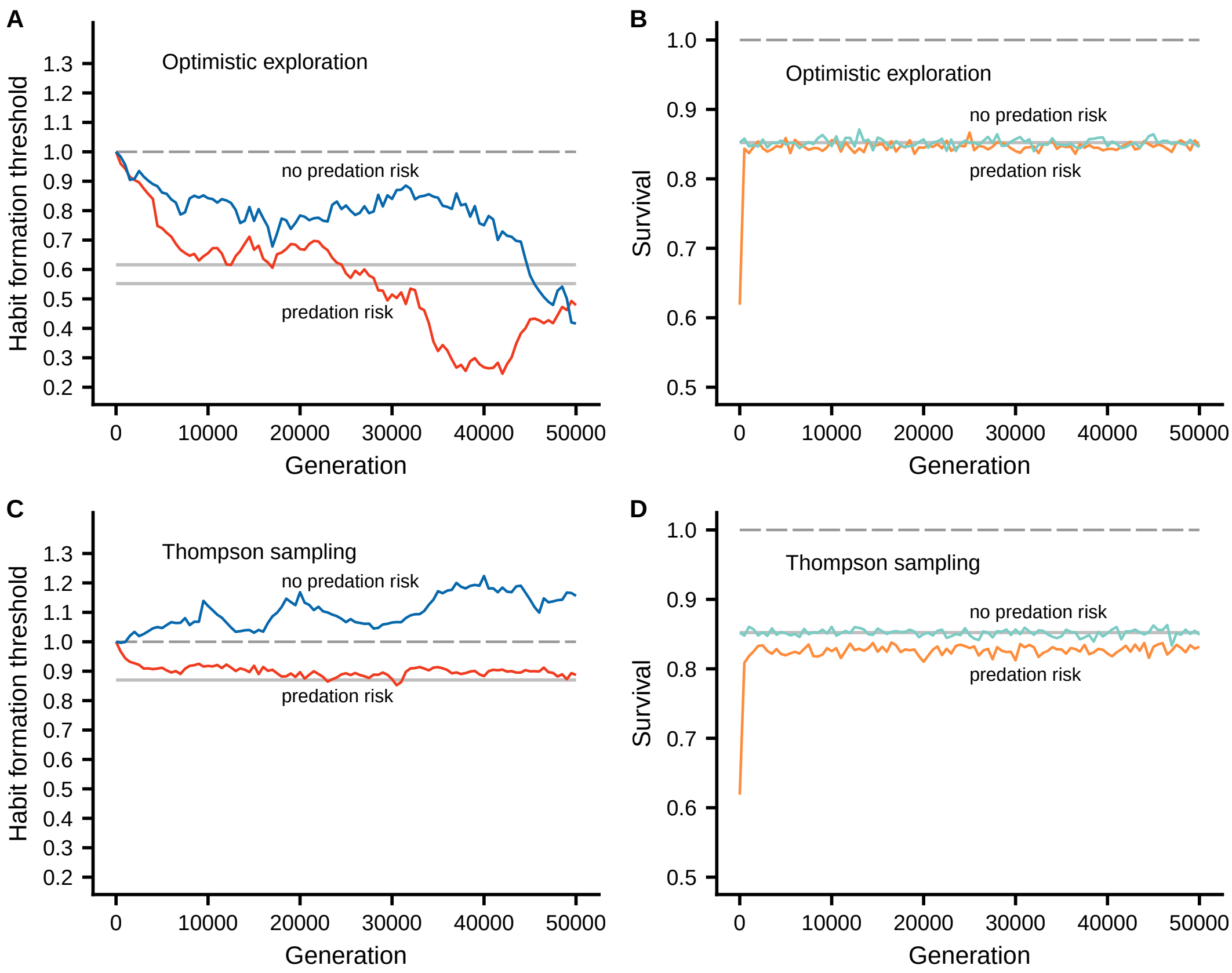

Figure S6: Illustration of evolutionary trajectories of population mean habit formation thresholds $\hat{h}$ and survival over 50,000 generations. Each panel shows a comparison between situations where habit formation influences predation risk (red/orange curves; $\mu_1 = 0.00032$) and where there is no influence (blue/green curves; $\mu_1 = 0$). The exploration method is OE in panels **(A)** and **(B)**, and TS in **(C)** and **(D)**. For OE, the parameters are as in as in Fig. 3C,D ($\mu_1 = 0.00032$) and Fig. 4C,D ($\mu_1 = 0$), and the trait values are kept fixed at these evolutionary equilibria, except that the trait $\hat{h}$ is allowed to evolve from a starting point of $\hat{h} = 1$. The gray horizontal lines in **(A)** indicate the evolutionary equilibria for $\hat{h}$, and the line in **(B)** gives the theoretically expected survival of 0.85 without predation risk effects. For TS, parameters and traits are similarly as in Fig. S3C,D and Fig. S4C,D. For the predation risk cases, note that the survival rapidly increases from the starting value of 0.62 in both **(B)** and **(D)** (orange curves), showing that there is stronger selection to achieve rapid enough habit formation such that there is habitual foraging in most trials. Note also that the no-predation-risk threshold $\hat{h}$ in **(C)** evolves to stay above $\hat{h} = 1$, so that habits do not form.

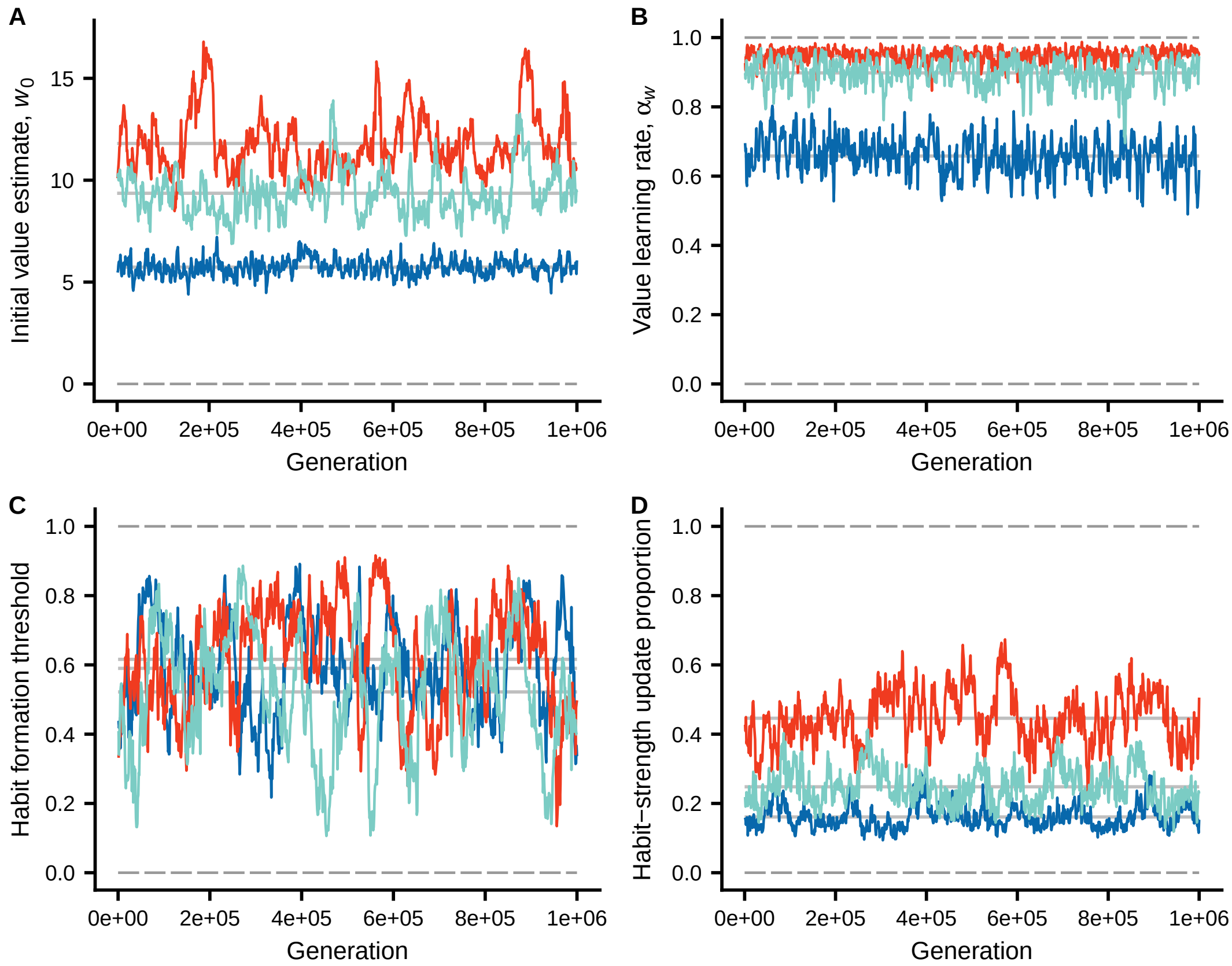

Figure S7: Illustration of evolutionary trajectories of population mean trait values over 10^6 generations, for the simulations shown in Figs. 2 and 3. Each panel shows trajectories for one trait: the initial value estimate w_0 in panel **(A)**, the value learning rate α_w in **(B)**, the habit-formation threshold $\hat{h}$ in **(C)**, and the habit-strength update proportion α_0 in **(D)**. The green lines are for the case of $\sigma_R = 0.25$ in Fig. 2, and the blue and red lines are for the cases $\sigma_R = 0.50$ and $\sigma_R = 0.10$ in Fig. 3. The horizontal light gray lines show the overall means, which are also given in the first three rows of Table S1; the standard deviations in the table are computed from the variation seen in the trajectories in this figure. Note that there is substantial variation over evolutionary time, in particular for the habit-formation thresholds in panel **(C)**. The overall mean values, and the general positions of the trajectories, still support the general conclusion from the results: the initial value estimates w_0 in **(A)** are larger in situations with less noisy rewards, the value learning rates α_w in **(B)** are higher in situations with less noisy rewards, and the rates of habit formation, measured by the habit-strength update proportion α_0 in **(D)**, are higher in situations with less noisy rewards.

Parameters and trait values from simulations

Table S1: Trait values: mean ± SD of population mean trait values from 1000 successive simulations, each over 1000 generations. There are 5 different cases of individual-based evolutionary simulations using OE presented in the main text. The parameters that vary between cases are noise in rewards (σ_R) and the influence of attention on predation risk (μ_1), while $\rho_0 = 0$, $\sigma_0 = 1$, $\mu_e = 8$, $\sigma_e = 1$, and two successive environments with 500 trials each hold for all cases.

$\sigma_R,\ \mu_1 \times 10^3$	w_0	α_w	α_0	α_h	$\hat{h}$	$\hat{e}$
0.25, 0.32	9.36 ± 1.11	0.90 ± 0.04	0.25 ± 0.05	0.14 ± 0.07	0.52 ± 0.17	0.52 ± 0.23
0.50, 0.32	5.73 ± 0.41	0.66 ± 0.05	0.16 ± 0.03	0.10 ± 0.04	0.59 ± 0.13	0.41 ± 0.24
0.10, 0.32	11.80 ± 1.49	0.95 ± 0.02	0.45 ± 0.08	0.28 ± 0.11	0.62 ± 0.15	0.62 ± 0.21
0.50, 0.0	5.14 ± 0.37	0.63 ± 0.05	0.07 ± 0.02	0.05 ± 0.02	0.73 ± 0.11	0.35 ± 0.25
0.10, 0.0	12.74 ± 2.09	0.94 ± 0.03	0.19 ± 0.08	0.11 ± 0.07	0.55 ± 0.19	0.49 ± 0.23

Table S2: Trait values: mean ± SD of population mean trait values from 1000 successive simulations, each over 1000 generations; for the last two simulations, with $\mu_1 = 0$, only 100 successive simulations were run. There are 5 different cases of individual-based evolutionary simulations using TS and presented in the supplements. The parameters that vary between cases are noise in rewards (σ_R) and the influence of attention on predation risk (μ_1), while $\rho_0 = 0$, $\sigma_0 = 1$, $\mu_e = 8$, $\sigma_e = 1$, and two successive environments with 500 trials each hold for all cases.

$\sigma_R,\ \mu_1 \times 10^3$	α_h	$\hat{h}$	$\hat{e}$
0.25, 0.32	0.08 ± 0.02	0.83 ± 0.05	0.57 ± 0.20
0.50, 0.32	0.09 ± 0.02	0.79 ± 0.05	0.46 ± 0.23
0.10, 0.32	0.08 ± 0.01	0.87 ± 0.04	0.38 ± 0.20
0.50, 0.0	–	> 1	0.14 ± 0.10
0.10, 0.0	–	> 1	0.25 ± 0.10

Summary of notation for the models

Table S3: Table of notation for the OE and TS models.

notation	definition or explanation
$\bar{R}_k$	expected reward from option k (log-normally distributed)
ρ_0	log-scale mean of distribution of the $\bar{R}_k$
σ_0	log-scale standard deviation of distribution of the $\bar{R}_k$
$R_k(t)$	reward from option k in trial t (log-normally distributed)
σ_R	log-scale standard deviation of distribution of $R_k(t)$ around $\bar{R}_k$
$a(t)$	attention to exploration and learning in trial t (0/1 variable)
μ_0, μ_1	parameters for predation mortality: $\mu_{\text{pred}}(t) = \mu_0 + \mu_1 a(t)$
$h_k(t)$	consistency of choice of option k in trial t; $0 \leq h_k(t) < 1$
α_h	rate of update of $h_k(t)$ (trait)
$\hat{h}$	habit formation threshold for the consistencies $h_k(t)$ (trait)
z_{e}	cue of environmental change (normally distributed)
$\mu_{\text{e}}, \sigma_{\text{e}}$	mean and SD of z_{e}; examples in main text: $\mu_{\text{e}} = 2, \sigma_{\text{e}} = 1$
p_{e}	probability of environmental change: $p_{\text{e}} = 1/(1 + \exp(-z_{\text{e}}))$
$\hat{e}$	threshold for individual to respond to p_{e} (trait)
$w_k(t)$	estimated reward value of option k in trial t
w_0	initial and reset value of w_k (OE trait)
α_w	value learning rate (OE trait)

Alternative mechanism for the breaking of habits

As mentioned in the main text, apart from the assumption of specific cues of environmental change, there are alternative ideas about the breaking of habits. One such idea is that a lowering of the rate of rewards below a threshold causes a transition from habitual foraging to exploration. Here we evaluate this idea, by introducing a modified model and then performing evolutionary simulations with this model, corresponding to the situations in Figs. 2 and 3 in the main text. The modified model uses OE and keeps the previous assumptions about environments and the formation of habits, as described in equations (1) to (7) in the main text, but removes the assumptions in equations (S1) and (S2) as well as the trait $\hat{e}$, which deal with the breaking of habits. The modified habit breaking mechanism is then as follows.

Individuals maintain an estimate $R_{\text{e}}(t)$ of the recent rate of rewards, which is updated through

$$R_{\text{e}}(t+1) = R_{\text{e}}(t) + \alpha_R \big(R(t) - R_{\text{e}}(t) \big), \tag{S6}$$

where $R(t)$ is the perceived reward in trial t and α_R is a rate of update of the estimate. The estimate is then used for the breaking of habits. If attention is off in trial t, so that foraging is habitual, the individual can break the habit if the estimated rate of reward R_{e} goes below a threshold $\hat{R}$:

$$R_{\mathrm{e}}(t) < \hat{R}. \tag{S7}$$

This turning on of attention is a full reset of the individual's state, including of any learned estimates of the value of feeding options. In the modified model, the threshold $\hat{R}$ is introduced as an additional evolving trait, replacing the previous $\hat{e}$. For simplicity, we assume a given value for the update rate α_R, putting $\alpha_R = 0.5$ in our simulations. To avoid the possibility of a rapid switching on and off of attention, we introduce the requirement that there has been at least $\hat{t}$ trials of habitual foraging before applying condition (S7). In the simulations we used $\hat{t} = 50$.

Comparing Fig. S8 with Fig. 2 in the main text, we see that there is qualitative similarity between the results, but also some notable differences. The most striking difference is perhaps that mean rewards and regrets in panel A, and even more the mean consistency and and attention in panel B, show oscillating patterns. The explanation is that a fraction of the individuals encounter an environment where all foraging options have mean rewards around or below the habit breaking threshold (note that each option in each environment is randomly generated). This causes these individuals to obtain estimates R_{e} below the threshold $\hat{R}$ for most trials during habitual foraging (this is the reason we introduced the requirement of at least $\hat{t}$ trials of habitual foraging), resulting in a switching on and off of habitual foraging. At the evolutionary equilibrium, the fraction that switches in this way is small, such that the mean survival in the population is similar in Fig. S8 to that in Fig. 2 in the main text.

From the example individual in panels C and D in Fig. S8, we see an additional difference: the habit breaking does not occur immediately in the new environment. In the example, after seven trials in the new environment the estimate R_{e} falls below the threshold, after which there is learning and then the forming of a new habit in trial 516.

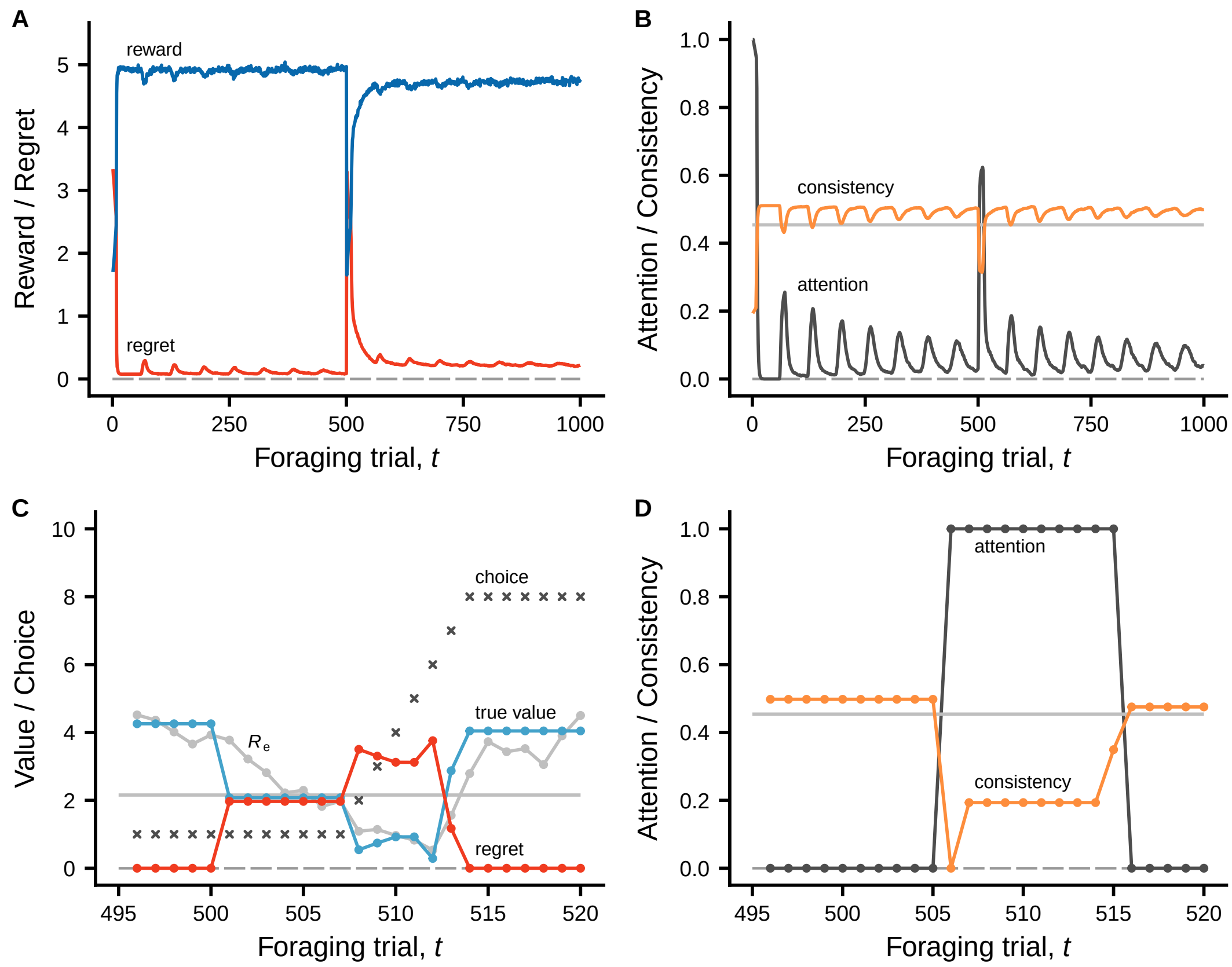

Figure S8: The forming and breaking of habits at an evolutionary equilibrium, similar to the situation in Fig. 2 in the main text, but for the modified model with habit breaking based on a threshold for an estimated rate of reward. As in that figure, there are eight feeding options, with expected reward values drawn from a log-normal distribution (log-scale mean $\rho_0 = 0$ and SD $\sigma_0 = 1$) and trial-to-trial random reward variation with $\sigma_R = 0.25$. There are a total of 4000 simulated individuals, with traits for optimistic exploration and habit formation and breaking at evolutionary equilibrium. Panel **(A)** shows the mean over all individuals of the reward in each trial (blue curve), together with the mean regret (red curve). The mean attention to foraging (dark gray) and the mean consistency of choice (orange) are in panel **(B)**. Individuals turn off attention to foraging and become habitual when consistency goes above an evolved threshold $\hat{h}$, indicated by a gray horizontal line. Panels **(C)** and **(D)** show an example of one individual, for the last 5 trials of the first environment and the first 20 trials of the second environment. The successive estimates R_e are shown as a light gray line with points, and the habit breaking threshold $\hat{R}$ as a light gray horizontal line in **(C)**. The successive choices are indicated by dark gray crosses, together with the corresponding expected (true) values w (blue-green) and regret values (red). The average survival over 1000 trials is around 0.83. Trait values: $w_0 = 9.91$, $\alpha_w = 0.97$, $\alpha_0 = 0.43$, $\alpha_h = 0.21$, $\hat{h} = 0.45$, $\hat{R} = 2.16$.

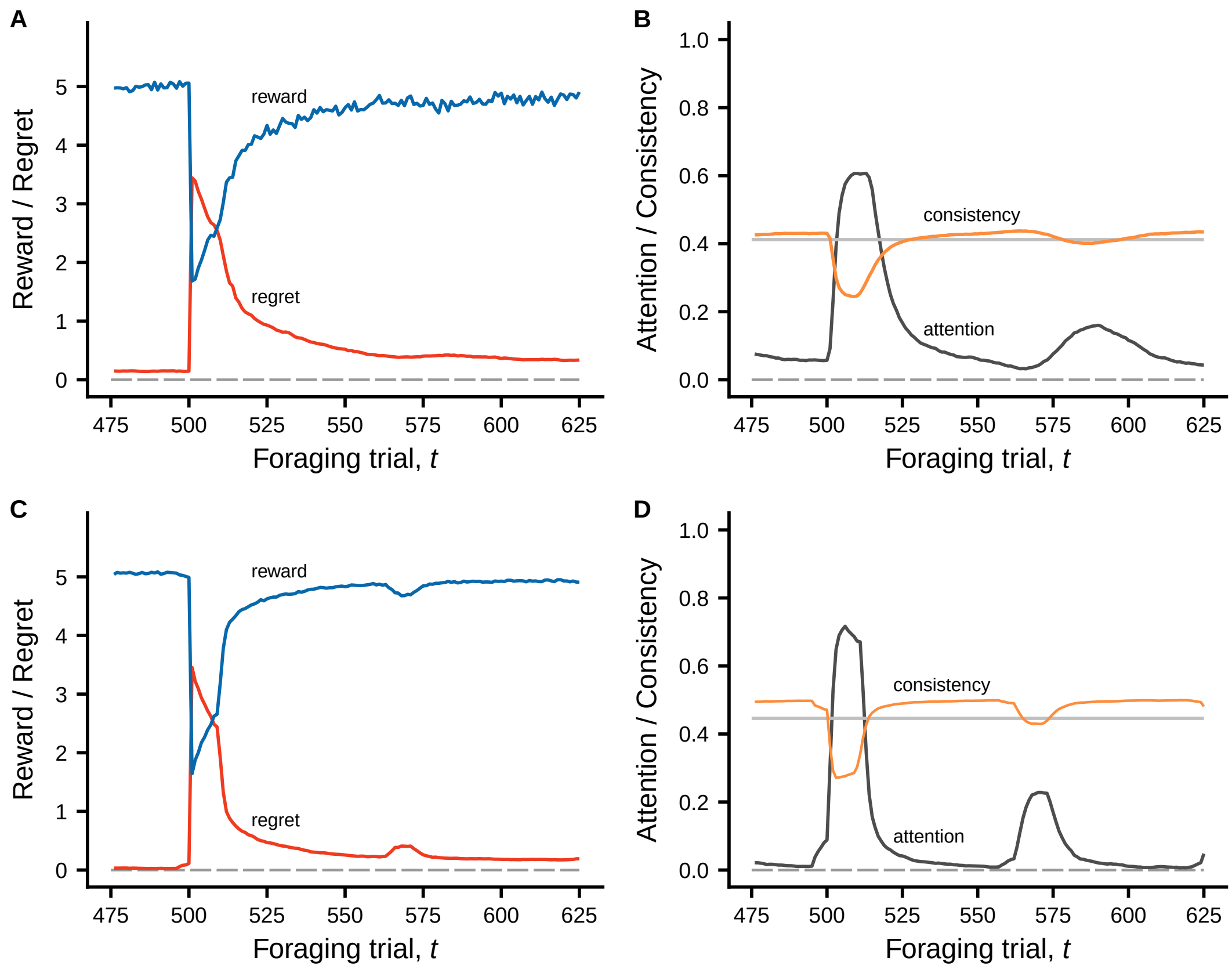

Figure S9: The influence of the predictability of rewards on the speed of habit formation, similar to the situation in Fig. 3 in the main text, but for the modified model with habit breaking based on a threshold for an estimated rate of reward. The figure shows means of reward, regret, attention, and choice consistency for evolutionary equilibria of traits of optimistic exploration and habit formation. The situations are similar to Fig. S8, except that the random variation of rewards for a given option is high ($\sigma_R = 0.50$) in panels **(A)** and **(B)**, and low ($\sigma_R = 0.10$) in panels **(C)** and **(D)**. The panels show trials around the time of environmental change at $t = 500$ trials. With less predictable rewards **(A, B)** habitual foraging takes longer to develop compared to the case with more predictable rewards **(C, D)**. Among the factors behind the difference, the consistency update rate α_h is lower in **(A, B)** than in **(C, D)**. At evolutionary equilibrium, the trait values for lower, higher predictability are $w_0 = 6.09, 11.88$, $\alpha_w = 0.44, 0.63$, $\alpha_h = 0.21, 0.34$, $\hat{h} = 0.69, 0.78$, $\hat{R} = 1.48, 2.66$, $\beta = 13.0, 11.9$.

Comparing Fig. S9 with Fig. 3 in the main text, there is again qualitative similarity between the results. There are also the same types of differences, in the form of oscillatory tendencies. For the case with the least predictability of rewards (panels A and B in Fig. S9), the regrets are noticeably higher at the start of a new environment in comparison with Fig. 3. A likely explanation is that in situations with fairly noisy

rewards, an unambiguous cue of a new environment, as in Fig. 3A,B, is considerably more efficient than using an estimate of recent rewards to detect a new environment, as in Fig. S9A,B.

References

Russo, D. J., Roy, B. V., Kazerouni, A., Osband, I., and Wen, Z. 2018. A tutorial on Thompson Sampling. *Foundations and Trends in Machine Learning*, 11(1):1–96.

Thompson, W. R. 1933. On the likelihood that one unknown probability exceeds another in view of the evidence of two samples. *Biometrika*, 25(3/4):285–294.
